# A reference genome of the Chinese hamster based on a hybrid assembly strategy

**DOI:** 10.1002/bit.26722

**Published:** 2018-05-29

**Authors:** Oliver Rupp, Madolyn L. MacDonald, Shangzhong Li, Heena Dhiman, Shawn Polson, Sven Griep, Kelley Heffner, Inmaculada Hernandez, Karina Brinkrolf, Vaibhav Jadhav, Mojtaba Samoudi, Haiping Hao, Brewster Kingham, Alexander Goesmann, Michael J. Betenbaugh, Nathan E. Lewis, Nicole Borth, Kelvin H. Lee

**Affiliations:** ^1^ Bioinformatics and Systems Biology Justus‐Liebig‐University Giessen Giessen Germany; ^2^ Department of Computer and Information Sciences University of Delaware Newark Delaware; ^3^ Delaware Biotechnology Institute Newark Delaware; ^4^ Department of Bioengineering University of California San Diego California; ^5^ Novo Nordisk Foundation Center for Biosustainability University of California San Diego California; ^6^ Austrian Center of Industrial Biotechnology Vienna Austria; ^7^ Chemical and Biomolecular Engineering Johns Hopkins University Baltimore Maryland; ^8^ Department of Biotechnology University of Natural Resources and Life Sciences Vienna Austria; ^9^ Department of Biorescources Fraunhofer Institute for Molecular Biology and Applied Ecology Giessen Germany; ^10^ Department of Pediatrics University of California San Diego California; ^11^ Johns Hopkins University Deep Sequencing and Microarray Core Johns Hopkins University Baltimore Maryland; ^12^ Department of Chemical and Biomolecular Engineering University of Delaware Newark Delaware

**Keywords:** assembly, biopharmaceuticals, Chinese hamster, genome

## Abstract

Accurate and complete genome sequences are essential in biotechnology to facilitate genome‐based cell engineering efforts. The current genome assemblies for *Cricetulus griseus*, the Chinese hamster, are fragmented and replete with gap sequences and misassemblies, consistent with most short‐read‐based assemblies. Here, we completely resequenced *C. griseus* using single molecule real time sequencing and merged this with Illumina‐based assemblies. This generated a more contiguous and complete genome assembly than either technology alone, reducing the number of scaffolds by >28‐fold, with 90% of the sequence in the 122 longest scaffolds. Most genes are now found in single scaffolds, including up‐ and downstream regulatory elements, enabling improved study of noncoding regions. With >95% of the gap sequence filled, important Chinese hamster ovary cell mutations have been detected in draft assembly gaps. This new assembly will be an invaluable resource for continued basic and pharmaceutical research.

## 1 INTRODUCTION

For decades, Chinese hamster ovary (CHO) cells have been the primary recombinant protein production host across the biopharmaceutical industry (Walsh, 2014). Characteristics, such as glycosylation, fast growth, and ease of genetic manipulation, help explain their prevalence. The history of CHO cells dates back to the 1950s, when ovarian connective tissue was harvested from the Chinese hamster and derivative cells spontaneously became immortal (Tjio, 1958). Since then, CHO has diverged into different adherent and suspension cell lines, such as CHO‐K1, CHO‐S, and CHO DG44 (Lewis et al., 2013). Their protein production capacity has been greatly improved through decades of refinements in bioprocessing strategies, media optimization, and engineering of transgenes and expression vectors. However, little engineering was done on the host cell itself, which remained poorly characterized for decades. Increasing demands on quantities of difficult‐to‐express‐proteins, protein quality, and time‐to‐market now require new strategies that involve cell engineering.

To facilitate CHO cell research and development, the community relies on published genomes for the CHO‐K1 cell line and the parent Chinese hamster, sequenced using short‐read Illumina technologies (Brinkrolf et al., 2013; Lewis et al., 2013; Xu et al., 2011; Yusufi et al., 2017). These resources have enhanced the use of transcriptomics, proteomics, genetic engineering, and other technologies (Kildegaard, Baycin‐Hizal, Lewis, & Betenbaugh, 2013; Lee, Grav, Lewis, & Faustrup Kildegaard, 2015; Richelle & Lewis, 2017) to understand and engineer desired traits in cells. However, to improve the accuracy in such endeavors, there is a need for genomic resources with a far more contiguous sequence and less pervasive gaps. The acquisition of such contiguous sequences is now possible with third‐generation sequencing technologies, such as single molecule real time (SMRT) sequencing technology (Eid et al., 2009), which provide mean read lengths that are more than an order of magnitude larger than earlier sequencing technologies. The reads can span repetitive elements, resulting in longer contigs and minimal gaps within scaffolds (Bickhart et al., 2017; Gordon et al., 2016; Jiao et al., 2017). This enables the routine assembly of mammalian genomes approaching the current quality of the human genome.

To obtain a higher quality reference assembly of the Chinese hamster, we have resequenced Chinese hamster liver tissue using long‐read SMRT technology at 45× coverage. Assemblies generated with Illumina or SMRT sequencing data were merged with the existing publicly available assemblies. Assembly merging yielded four candidate assemblies, which were evaluated for completeness and quality using 80 assembly metrics. Merging the platform‐specific assemblies results in a more contiguous, accurate, and complete genome assembly than using either technology alone. The final assembly presented is the most complete Chinese hamster genome to date, with the number of scaffolds reduced to fewer than 3%–6% the number in earlier works, and the mean contig length 16 to 29 times longer. The new genome shows substantial improvement in gene completeness and the extent of flanking noncoding DNA, thereby enabling the identification of promoters and enhancers. Finally, 95% of the sequence gaps were filled, exposing hundreds of cell line‐specific mutations in coding regions of the genome for several CHO cell lines. For example, an important single nucleotide polymorphism (SNP) in the glycosyltransferase, xylosyltransferase 2 (Xylt2), which impacts glycosylation and which was hidden in gaps in previous assemblies, can now be detected. Thus, this resource will serve as an important reference genome for researchers across the biotechnology industry and scientific community.

## 2 MATERIALS AND METHODS

### 2.1 Sequencing

#### 2.1.1 Illumina sequencing

Short‐read data from Chinese hamster liver tissue were generated using Illumina’s sequencing technology in two previously published studies. These included chromosome separated paired‐end libraries and mate‐pair short‐read data (Brinkrolf et al., 2013), and whole‐genome libraries with different insert sizes (Lewis et al., 2013). The size and coverage of sequencing libraries are shown in Supporting Information Table S8.

#### 2.1.2 Pacific biosciences SMRT sequencing

##### Preparation of Chinese hamster tissue

Five female Chinese hamsters (strain 17 A/gy) were raised under certified conditions. At 10 weeks of age, the individuals were euthanized by CO_2_ asphyxiation and verified by puncture wound to the abdomen. Livers were removed and cut into multiple pieces, flash frozen in liquid nitrogen, and stored at −80°C until further processing.

##### High‐molecular‐weight genomic DNA extraction

High‐molecular‐weight (HMW) genomic DNA extraction and purification from randomized liver samples were performed using the MagAttract HMW DNA Kit (Qiagen Inc., Venlo, Netherlands) as per the manufacturer’s instructions. HMW DNA was confirmed using a Fragment Analyzer (Advanced Analytical Technologies Inc., Ankeny, IA).

##### SMRT library preparation from genomic DNA samples

HMW DNA (10 µg aliquots) were converted into SMRTbell templates using the Pacific Biosciences RS DNA Template Preparation Kit 1.0 (Pacific Biosciences, Menlo Park, CA) as per the manufacturer’s instructions. In summary, samples were end‐repaired and ligated to blunt adapters. Exonuclease treatment was performed to remove unligated adapters and damaged DNA fragments. Samples were purified using 0.6× AMPureXP beads (Beckman Coulter Inc., Brea, CA). The purified SMRTbell libraries were eluted in 10 µl of elution buffer. Eluted SMRTbell libraries were size‐selected on the BluePippin (Sage Science Inc., Beverly, MA) to eliminate library fragments below 5 kb. Final library quantification and sizing was carried out on a Fragment Analyzer (Advanced Analytical Technologies Inc.) using 1 µl of library. SMRTbell templates were aliquoted, shipped, and prepared for sequencing at the University of Delaware Sequencing & Genotyping Center and the Johns Hopkins University Deep Sequencing and Microarray Core.

##### SMRT sequencing on the Pacific Biosciences RSII

The amount of primer and polymerase required for the binding reaction was determined using the SMRTbell concentration and library insert size. Primers were annealed and polymerase was bound to SMRTbell templates using the DNA/Polymerase Binding Kit P5 and P6 (Pacific Biosciences). Sequencing was performed using DNA sequencing reagent C3 and C4 (Pacific Biosciences) with Pacific Biosciences RSII sequencers and SMRT Cell V3 (Pacific Biosciences) at the University of Delaware Sequencing & Genotyping Center (DBI) and the Johns Hopkins University Deep Sequencing and Microarray Core (JHU). RSII loading efficiency was optimized for each individual library utilizing a standardized titration protocol. Over the course of the project, data capture time for the sequencing runs was initially set at 4 hr. This was extended to 6 hr after software upgrades.

##### SMRT data metrics

The two sequencing centers ran a total of 202 SMRT cells (92 DBI, 110 JHU). A total of 65 SMRT cells were run using P5/C3 chemistry, whereas 137 SMRT cells were run using P6/C4 chemistry. After filtering and adapter trimming, a total yield of 107.45 Gb was generated from 13.49 million sequence reads or approximately 45× coverage of the 2.4 Gb genome. The mean read length calculated from all generated reads was 11.55 kb. N50 read length calculated from all generated reads was 15.9 kb.

#### 2.1.3 SMRT read error‐correction

Before assembly, SMRT reads were error‐corrected (SMRT reads have 15% errors precorrection). As insufficient SMRT coverage was obtained for self‐correction of SMRT reads, we used Illumina paired‐end reads (Brinkrolf et al., 2013; Lewis et al., 2013) for SMRT read error correction. The reads were preprocessed with the ALLPATHS‐LG error‐correction module for fragment libraries (Gnerre et al., 2011). The reads from the same pair are joined, possible gaps are filled, and the read is error‐corrected, resulting in a longer, single, and error‐free read. Two different tools for error correction were tested with different parameters: proovread (Hackl, Hedrich, Schultz, & Förste, 2014) and LoRDEC (Salmela & Rivals, 2014). The tools were tested separately and in combination. The best results were achieved when, in the first step, proovread was run on the initial reads with a single iteration on the complete Illumina reads. All Illumina reads were mapped to all SMRT reads (allowing for multimappings) using the modified version of BWA in the proovread tool. Then, the bam2cns algorithm in proovread was applied to correct the reads based on the majority decision of the Illumina mappings. In the second step, the proovread results were further processed with LoRDEC. Using the corrected reads, LoRDEC created a de Bruijn graph from the Illumina reads, mapped the nodes (k‐mers of size 85) to the SMRT reads, and corrected the unmapped regions following a path in the de Bruijn graph. See Supporting Information Text and Figures S10‐S11 for more details.

### 2.2 Genome size estimation

Genome size was estimated by the k‐mer frequency of the Illumina read data using (1) all Illumina whole‐genome paired‐end libraries with an insert‐size of 500, (2) the libraries with an insert size of 800, and (3) a combination of sets one and two. Jellyfish (Marçais & Kingsford, 2011) was used to count the frequencies for k‐mers of 17, 25, and 31. The GCE tool (Liu et al., 2013) was used to estimate the genome size.

### 2.3 Genome assembly

**Figure 1:**
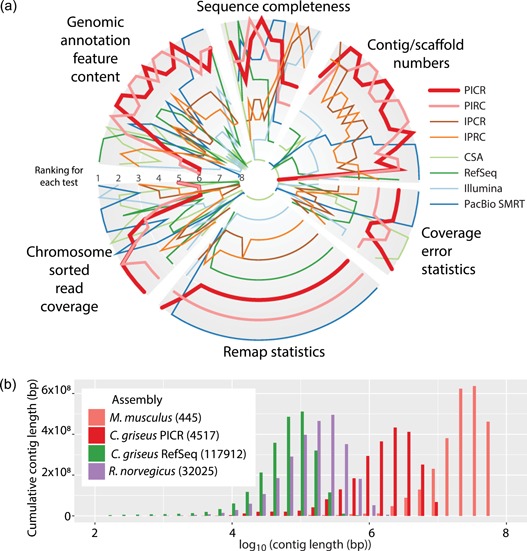
The PICR assembly ranked against other mammalian assemblies. (a) The PICR assembly was compared with other candidate assemblies of *Cricetulus griseus* based on 80 different assembly metrics. This shows for each test how the assemblies compare. The best assembly for each test is plotted on the outer rim, whereas the worst is near the center. Eighty tests were defined (see Supporting Information Table S3) in six different categories. On average, the PICR assembly was the most highly ranked, with the PIRC assembly closely following. (b) Weighted histogram of the contig lengths for the PICR assembly (red) compared with the Ensemble mouse (salmon), rat (purple), and the previous Chinese hamster RefSeq assemblies (green) [Color figure can be viewed at wileyonlinelibrary.com]

The final genome assembly was conducted in two stages. In the first stage, four different assemblies were built with different tools and library combinations using the raw Illumina or the error‐corrected SMRT reads. In the second stage, the four primary assemblies were iteratively merged in four different orders using the Metassembler tool (see Supporting Information Figure S8) (Wences & Schatz, 2015). Various quality metrics (Figure 1) were used to assess the quality of the eight assemblies (four primary assemblies and four metassemblies). These metrics were further used to rank the assemblies and select the assembly with the best overall rank. Finally, the PICR was used as the reference assembly after polishing by correcting the single detected misassembly and minor gap filling from the PIRC assembly (see Supporting Information).

#### 2.3.1 Primary assemblies

##### Assembly 1: Illumina‐based chromosome‐sorted assembly

The ten chromosome sorted libraries were assembled separately, including the whole‐genome mate‐pair library to each assembly, with the ALLPATHS‐LG tool (Gnerre et al., 2011). The resulting scaffolds were filtered for possible contaminations of other chromosomes. The final assembly has been previously published (Brinkrolf et al., 2013) and is available at the NCBI assembly archive (accession: GCA_000448345.1).

##### Assembly 2: Whole‐genome Illumina assembly (RefSeq)

The RefSeq reference genome of the Chinese hamster is based on the SOAPdenovo2 (Luo et al., 2012) assembly (Lewis et al., 2013). The different paired‐end and mate‐pair Illumina libraries were assembled using SOAPdenovo2 (Luo et al., 2012). The assembly is accessible at the NCBI assembly archive (accession: GCA_000419365.1).

##### Assembly 3: Whole‐genome and chromosome‐sorted assembly (Illumina)

Sequence data originating from the published chromosome‐sorted Illumina libraries and whole‐genome Illumina libraries (Brinkrolf et al., 2013; Lewis et al., 2013) were combined and assembled with the ALLPATHS‐LG tool (version 51927; Gnerre et al., 2011).

##### Assembly 4: Pacific Biosciences SMRT assembly

The ALLPATHS‐LG tool was used to merge and error‐correct overlapping paired‐end Illumina reads, and these reads were further extracted and converted into FASTA format to aid in the SMRT error‐correction process. The error‐corrected SMRT reads were assembled following the HGAP‐3 pipeline (Chin et al., 2013) without the error‐correction step. For better control over the workflow, we used the customizable makefile‐based smrtmake workflow (smr, 2016).

#### 2.3.2 Merged assemblies

The four primary assemblies were iteratively merged with the Metassembler (Wences & Schatz, 2015) tool. For each meta‐assembly, one assembly is selected as the primary assembly. The scaffolds of a second assembly are subsequently mapped to the primary scaffolds using NUCmer (Kurtz et al., 2004). A CE‐statistic, based on the distance of mate‐pair reads, is computed for both assemblies. Primary scaffolds are joined and gaps are closed with the sequence of the second assembly. If the CE statistics of the primary scaffolds indicate potential errors, the sequence in this area is replaced by the sequence in the second assembly. The resulting scaffolds are then used as primary scaffolds for the next iteration. Changes to the default parameters were applied for the merging step (asseMerge). The minimal range for finding links between scaffolds was increased to 50,000 and the minimal coverage of the secondary scaffold was lowered to one. The minimal gap size for closure was lowered to one (asseMerge ‐e 50000 ‐L 1 ‐t 1). The order in which the assemblies are merged influences the result of the final meta‐assembly, and four different orders were tested (see Table 2).

### 2.4 Chromosome assignment

Scaffolds were assigned to chromosomes using chromosome‐sorted library coverage, computed for 1 kb regions. Specifically, for each 1 kb region of each scaffold, the coverage of each chromosome library was computed. If at least 90% of the 1 kb region of a scaffold showed a normalized coverage between 0.5 and 2 of the same chromosome, the scaffold was assigned to this chromosome. Scaffolds assigned to the pooled chromosome 9 and 10 library and all unassigned scaffolds were mapped to the mouse genome using NUCmer (Kurtz et al., 2004). Yang, O’Brien, and Ferguson‐Smith (2000) and Wlaschin & Hu (2007) described the localization of the hamster chromosomes on the mouse chromosomes. This information was used to assign the mapped scaffolds to a chromosome by manually comparing the mapped position with the localization from Yang et al. (2000) and Wlaschin & Hu (2007).

### 2.5 Gene prediction and annotation

#### 2.5.1 Gene annotation

Annotation of the PICR and IPCR metassemblies was completed using Maker v2.31.8. Chinese hamster ESTs (40 million reads) from SRA (SRR823966) were assembled using Trinity v2.0.6 (Grabherr et al., 2011). The resulting transcripts were aligned to the previously published hamster transcriptome assembly (Lewis et al., 2013), which had used Trinity v. r2011‐08‐20. NUCmer (Kurtz et al., 2004) was used for the alignment with default parameters. A total of 91,027 transcripts were found in both transcriptomes and used as evidence for gene prediction within Maker. In addition, all proteins from the 2014 RefSeq annotation (GCF000419365.1) of the hamster genome were used as evidence. Comparisons with mouse, rat, and the RefSeq hamster annotations are provided in Supporting Information Table S9 and Figures S12, S13, S14, S15, S16, and S17.

Repeat masking was done within the Maker pipeline. To identify repeat regions, we used Repeat‐Masker version open‐4.0.6 (Smit, Hubley, & Green, 2015). Dfam v2.0 (2015‐09‐23), a database of eukaryotic transposable element and other repetitive DNA sequence alignments, and the RepeatMasker database (release 2015‐08‐07, derived from RepBase v20.08). Once repeat masking was completed, BLAST v2.2.28 (Camacho et al., 2009) and exonerate v2.2.0 (Slater & Birney, 2005) were run within Maker for evidence‐based alignments and SNAP v2006‐07‐28 (Korf, 2004) and Augustus v3.2.2 (Keller, Kollmar, Stanke, & Waack, 2011) for ab initio gene prediction.

The resulting annotation only included genes with more than one type of evidence supporting the prediction, that is, both an ab initio prediction and an evidence‐based alignment. Functional annotation of Maker’s output was done as described in “Support Protocol 3: Assigning putative gene function” of “Genome Annotation and Curation Using MAKER and MAKER‐P” (Campbell, Holt, Moore, & Yandell, 2014). BLAST was used (*e*‐value  < 0.001) for each predicted gene against the Swiss‐Prot release‐2016‐02 database, where the best hit was used as the putative function of that gene.

Further comparisons were made based on the NCBI annotation pipeline, as detailed in the supplementary text and Supporting Information Tables S10–S11.

### 2.6 Gap analysis

#### 2.6.1 Identification of the filled‐gap sequence

We aligned the Chinese hamster RefSeq genome sequence to the PICR genome sequence using NUCmer (Kurtz et al., 2004) to identify gap sequence (see Supporting Information Figure S9). Briefly, NUCmer clusters a set of maximally exact matches as an anchor and then extends alignments between the clustered matches. Gaps are represented using letters N in the genome, and since they differ between the RefSeq and PICR meta‐assembly, the MUMmer alignments stop at gaps larger than 89 bp (base pairs). This means that if two fragments that flank both ends of a gap are found on the same PICR scaffold in the same orientation, the sequence between the two matches corresponds to the sequence of the gap. Since sequence errors may occur near gap regions, we consider matches flanking a gap if the distance between the fragment and the gap is less than 10 bp. When the gap is shorter than 90 bp, MUMmer clusters the gap together with the two matches on both ends and only reports the merged long fragment as mapping. In this case, we first used the show‐aligns method in MUMmer to output the alignment details between the RefSeq hamster and PICR, and then we extracted the corresponding gap sequence by parsing the alignments. The gap analysis was performed using PICR and RefSeq hamster assembly, except the gap in the Xylt2 gene, which was visualized using the RefSeq CHO‐K1 genome assembly.

#### 2.6.2 Identification of genes with gaps and mutations

We called variants in whole‐genome resequencing data from various CHO cell lines (Feichtinger et al., 2016; Lewis et al., 2013; van Wijk et al., 2017). GATK v3.5 (Auwera et al., 2013; DePristo et al., 2011; McKenna et al., 2010) was used with the GATK manual‐recommended parameters. We also called variants using the reads from the RefSeq assembly project (Lewis et al., 2013) to identify and filter false‐positive variants. Pybedtools (Dale, Pedersen, & Quinlan, 2011; Quinlan & Hall, 2010) identified genes with gaps in their coding regions. Gene ontology (GO) term analysis was performed using DAVID (Huang, Sherman, & Lempicki, 2009a, 2009b).

First, to identify classes of genes with gaps in the RefSeq assembly, we mapped all hamster genes to their human homologs. The functional enrichment analysis for all the 2,252 genes with coding gap regions was performed using the human genes with hamster homologs as the background. Second, to identify classes of genes with a higher frequency of mutations in gaps, we looked for over‐representation of the 132 genes with variants in coding gaps, while using the 2,252 gap‐filled genes as the background. GO terms with a *p*‐value smaller than 0.01 were visualized using REViGO (Supek, Bošnjak, Škunca, & Šmuc, 2011). Code for the gap analysis can be acquired here https://github.com/LewisLabUCSD/assembly_gaps.

## 3 RESULTS

### 3.1 Platform‐specific assemblies of the Chinese hamster genome

#### 3.1.1 Pooled Illumina assembly

In two independent previous attempts, the Chinese hamster genome was generated using Illumina sequencing from DNA isolated from liver tissue acquired from the same hamster colony as that used for deriving CHO cells in 1957 (Brinkrolf et al., 2013; Lewis et al., 2013). The current RefSeq assembly originated from whole‐genome libraries with varying insert sizes (Lewis et al., 2013). A second assembly (chromosome‐sorted assembly [CSA]) using chromosome sorted sequencing libraries is also publicly available (Brinkrolf et al., 2013). The different libraries combined yielded about two billion read pairs with read lengths from 99 to 150 bp, in total 442.22 Gb (see Supporting Information for details). K‐mer‐based genome size estimations of different libraries and k‐mers ranged between 2.55 Gb and 2.75 Gb.

We de novo assembled the pooled Illumina reads from both previous assemblies using ALLPATHS‐LG. This Illumina assembly contained 2.39 Gb of scaffolds with 2.66% gaps. The scaffold N50 number (the minimal number of scaffolds needed to cover 50% of the assembled genome) was 128, with an N50 length (length of the smallest N50 scaffold) of 5.95 Mb (Table 1), which was much greater than the previously published assemblies.

**Table 1:** Assembly metrics of the Illumina scaffolds and PacBio SMRT curated assembly compared with the previously published assemblies

	RefSeq (Lewis et al., 2013)	CSA (Brinkrolf et al., 2013)	Pooled Illumina scaffolds	Curated PacBio SMRT contigs
Scaffolds (No.)	52,710	28,749	17,373	1,659
Length (Gb)	2.36	2.33	2.39	2.31
Min length (bp)	201	830	898	100,560
Max length (Mb)	8.32	14.66	25.84	16.08
Mean length (kb)	44.78	81.14	137.45	1394.69
Median length (bp)	363	1,927	2,063	693,156
N50 length (kb)	1558.30	1236.52	5951.71	2906.73
N50 (No.)	450	501	128	223
N90 length (kb)	395.29	180.69	1003.29	623.9
N90 (No.)	1,558	2,251	468	884
Total N gaps (No.)	166,152	290,660	110,314	0
Total N (%)	2.49	10.45	2.66	0

*Note*. CSA, chromosome‐sorted assembly; PacBio SMRT, Pacific Biosciences SMRT assembly; N, undefined base in scaffolds.

#### 3.1.2 Pacific Biosciences SMRT assembly sequencing assembly

Pacific Biosciences SMRT (PacBio SMRT) sequencing yielded a 107.45 Gb total sequence from 13.49 million subreads, corresponding to ∼45× coverage of the 2.4 Gb genome (after filtering and adapter trimming). Pooled and corrected Illumina reads were used to correct sequencing errors of the SMRT reads. Specifically, overlapping paired‐end reads were merged and error‐corrected as part of the ALLPATHS‐LG (Gnerre et al., 2011) assembly process. This created about 836 million single reads, with a mean size of 171 bp and 143.75 Gb total. These were reused in the SMRT error‐correction, which was done in two steps using proovread (Hackl, Hedrich, Schultz, & Förster, 2014) and LoRDEC (Salmela & Rivals, 2014), leading to a reduction in the indel‐ratio (the number of indels divided by the number of matches in the alignments against the Illumina contigs) from 0.18 to 0.04. SMRT reads were assembled using HGAP (Chin et al., 2013), resulting in the assembly hereafter referred as the PacBio SMRT assembly. After removal of duplicate contigs (see Supporting Information Table S1), the assembly resulted in 2.3 Gb of nonredundant sequence with an N50 scaffold number of 223 and an N50 size of 2.9 Mb (Table 1).

### 3.2 A highly contiguous meta‐assembly is obtained by merging draft assemblies

Recent studies have highlighted the improvements in SMRT‐only assemblies compared with Illumina‐only assemblies (Bickhart et al., 2017; Gordon et al., 2016; Jiao et al., 2017; Shi et al., 2016; Zhang et al., 2016). Here, we found that both the pooled Illumina assembly (with mixed read length) and the PacBio SMRT‐only assembly resulted in substantially improved assembly statistics compared with the two published hamster genome assemblies (Table 1), with an order of magnitude fewer scaffolds and 2 to 4 times larger N50 values. However, the longer PacBio SMRT reads and the larger Illumina insert libraries should provide unique strengths that can be captured through assembly merging. Therefore, we aligned the scaffolds and contigs from four independent assemblies: the Illumina‐based CSA (Brinkrolf et al., 2013), the RefSeq assembly (Lewis et al., 2013), the pooled Illumina assembly developed here, and our de novo uncurated PacBio SMRT assembly. The Metassembler tool (Wences & Schatz, 2015) uses the first assembly provided as the base and subsequently merges additional assemblies. The tool was applied to the four assemblies using four different orders of merging, resulting in four different metassemblies, as shown in Table 2.

**Table 2:** Four different orders were used to merge the four initial assemblies with the Metassembler tool, where PICR starts with the PacBio SMRT assembly, after which the Illumina assembly is merged into it, followed by the CSA assembly and the RefSeq assembly

Base assembly	Added in Step 1	Step 2	Step 3	Name
PacBio SMRT	Illumina	CSA	RefSeq	PICR
PacBio SMRT	Illumina	RefSeq	CSA	PIRC
Illumina	PacBio SMRT	CSA	RefSeq	IPCR
Illumina	PacBio SMRT	RefSeq	CSA	IPRC

*Note*. CSA, chromosome‐sorted assembly; PacBio SMRT, Pacific Biosciences SMRT assembly; SMRT, single molecule real time.

All metassemblies showed considerable improvement over all initial draft assemblies (Table 3), with far fewer N50 scaffolds (only 32–34 compared with 223 for the PacBio SMRT and 128–501 for the Illumina‐based assemblies), and a significant decrease in the gap sequence compared with the Illumina‐only assemblies. Improvements in many metrics in all the intermediate merging stages show that all four initial draft assemblies contribute toward the improvement of the final assemblies (Supporting Information Figure S5). However, the metassemblies starting with the PacBio SMRT assembly outperformed the ones starting with the Illumina assembly in almost all metrics.

**Table 3:** Assembly metrics of the four merged assemblies

	PICR	PIRC	IPCR	IPRC
Scaffolds (No.)	1,829	1,825	2,317	2,304
Length (Gb)	2.37	2.37	2.36	2.36
Min length (bp)	568	568	915	915
Max length (Mb)	80.58	80.58	66.35	66.35
Mean length (kb)	1295.21	1298.43	1019.33	1024.64
Median length (bp)	37,019	38,181	13,201	14,241
N50 length (kb)	20188.72	19582.71	21744.88	21262.79
N50 (No.)	32	33	33	34
N90 length (kb)	4400.57	4422.38	3545.61	3650.27
N90 (No.)	121	122	122	122
Total N gaps (No.)	3,237	3,250	72,528	72,536
Total Ns (%)	0.12	0.12	1.13	1.13

*Note*. N, undefined base in scaffolds.

To validate the accuracy of assembly, chromosome‐separated sequencing libraries (Brinkrolf et al., 2013) were aligned to the scaffolds. Misassemblies can be easily identified by decreased read coverage from one chromosome and a rise in coverage from another (Supporting Information Figure S1). Manual inspection of all scaffolds larger than 1 Mb showed only one scaffold with a clear misassembly in the PacBio SMRT‐starting (PICR and PIRC) metassemblies and 11 in the metassemblies starting with Illumina scaffolds (IPCR and IPRC), whereas the current RefSeq assembly has >24 (Supporting Information Figure S2). Inspection of the chromosome coverage at the error region (Supporting Information Figure S3) showed a 30 kb region that contained low and mixed coverage, along with scaffolding gaps. This region was manually cut, and two new scaffolds were created. Ultimately, 96.6% of the sequence could be unambiguously assigned to a specific chromosome (Supporting Information Table S2).

### 3.3 The best assembly is identified using 80 assembly metrics

To quantify and compare the quality of our eight assemblies (including the four initial assemblies and the four metassemblies), we computed 80 different metrics (see Supporting Information), split into six classes covering different aspects of an assembly (Figure 1a; Supporting Information Figures S4 and S5 and Table S3), and ranked the assemblies for each class individually. The PICR meta‐assembly had the best overall rank in four of the six classes, followed by PIRC with two best overall ranks. Based on this evaluation, PICR was chosen for further analyses.

The PICR meta‐assembly has substantially longer contigs (contiguous sequences with “N”‐regions smaller than 100 bp) than the previous RefSeq assembly and even assemblies of some model organisms, such as the rat (*Rattus norvegicus*, assembly Rnor_6.0). In addition, PICR is approaching the continuity observed in the murine reference assembly (*Mus musculus*, assembly GRCm38.p5; Figure 1b and Supporting Information).

### 3.4 Polishing the final assembly

#### 3.4.1 Chromosomes are assigned using reads from flow‐sorted DNA

To assign each scaffold to a chromosome, we aligned all chromosome‐separated reads to the PICR meta‐assembly. 307 scaffolds were uniquely assigned to a chromosome, accounting for 94% of the genome (or 2.23 Gb). Unassigned scaffolds and scaffolds assigned to the unseparated hamster chromosome 9 and 10 library were instead mapped to the mouse genome. Scaffolds that could be aligned uniquely were assigned to a hamster chromosome based on published hamster chromosome localization (Wlaschin & Hu, 2007; Yang et al., 2000). Fifteen scaffolds (18.79 Mb) could be assigned to chromosome 9 and 2 scaffolds (32.58 Mb) to chromosome 10. A detailed list of assigned scaffold numbers and sizes is shown in Supporting Information Table S2. The final PICR assembly and the associated raw PacBio SMRT sequencing read data are available under NCBI BioProject PRJNA389969. The existing Illumina assemblies are available under NCBI BioProjects PRJNA167053 (RefSeq) and PRJNA189319 (CSA). Illumina sequencing data for BioProject PRJNA167053 are available from the Sequence Read Archive under SRP020466.

#### 3.4.2 Repeat masking, gene prediction, and annotation

We annotated the PICR and IPCR metassemblies using the Maker annotation tool (Holt & Yandell, 2011; Table 4 and Supporting Information Table S4). Due to the similarity of the PICR and PIRC assemblies, we decided to compare the annotation of PICR and IPCR. This comparison demonstrated the impact of using assemblies built from different sequencing methods as the primary assembly in Meta assembler. Repeat‐masker (Smit, Hubley, & Green, 2015) masked approximately 5.5 million repeats in PICR and 5.7 million in IPCR (Supporting Information Table S5).

**Table 4:** Gene and transcript information from the Maker annotation of the PICR and IPCR genome assemblies

Assembly		
All genes	PICR	IPCR
Gene count	24,686	23,410
Transcript count	24,948	23,656
Transcripts per gene	1.01	1.01
Average length transcript	17615.04	18089.17
Total length transcript	439,460,104	427,917,413
Average coding length	1324.93	1316.11
Total coding length	33,054,355	31,133,905
Average exons per transcript	7.49	7.54
Total exons	186,939	178,277
Complete transcripts		
Transcript count	18,476	17,557
Total exons	138,358	131,262
Incomplete transcripts		
Transcript count	6,472	6,099
Total exons	48,581	47,015

The Maker annotation yielded ∼1,300 more genes and transcripts in PICR than in IPCR. Functional annotations were assigned for 23,153 transcripts/proteins in PICR, but only 21,839 transcripts/proteins. in IPCR. The annotations of PICR and IPCR demonstrate that beginning assembly merging with the PacBio SMRT assembly, rather than the Illumina assembly, led to the identification and functional annotation of more genes.

The predicted proteins from PICR were searched using BLAST (*e*‐value ≤ 0.001) against the proteins from IPCR and vice versa to compare the annotation of the two assemblies. A total of 24,578 proteins in PICR have a BLAST hit in IPCR and 22,970 of these proteins have a functional annotation assigned from the top BLAST hit against the Swiss‐Prot database compared with 23,420 proteins in IPCR with a BLAST hit in PICR.

Analysis of the 236 proteins in IPCR, but not PICR, showed that most were not functionally annotated or were duplicates or isoforms of genes in PICR. Some proteins unique to the IPCR assembly include the protease carboxypeptidase Q (Cpq), the histone H3 threonine kinase haspin (Gsg2), the antioxidant sulfiredoxin‐1 (Srxn1), and the possible ortholog of DNA‐directed RNA polymerase III subunit RPC9 (Crcp). Analysis of the 367 proteins in PICR, but not IPCR, showed that about half were not functionally annotated. Proteins of interest unique to the PICR meta‐assembly include posphatidylglycerophosphate (pgp or pgs1), which is involved in phospholipid biosynthesis in mammalian cells (Kawasaki et al., 1999), and two DNA repair‐related proteins: breast cancer type 1 susceptibility protein (Brca1) and nonhomologous end‐joining factor 1 (NHEJ1). In addition, Bcl‐2‐like protein 10 (Bcl2l10), a signaling molecule involved in apoptosis, and stress‐associated endoplasmic reticulum protein 1 (Serp1) are both in PICR, but not IPCR. MicroRNAs targeting these two proteins in CHO cells have been developed (Jadhav et al., 2013).

#### 3.4.3 The PICR meta‐assembly has more contiguous genes and noncoding regulatory elements

**Figure 2:**
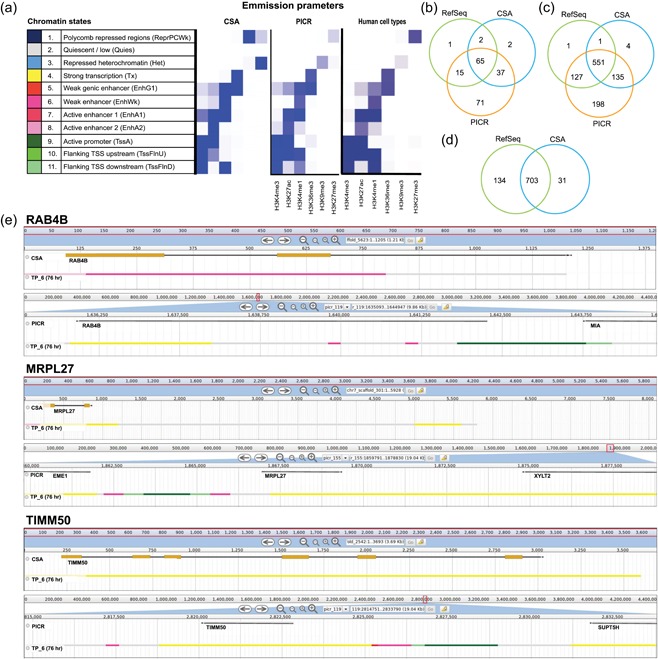
Importance of correct assembly of genes and noncoding regions. (a) Chromatin states defined by histone marks: Left: histone marks for CSA assembly (Brinkrolf et al., 2013; Feichtinger et al., 2016); center: histone marks for PICR assembly; right: histone marks from the Human Epigenome Project (Kundaje et al., 2015). (b) A total of 1,538 genes associated with mitochondria were blasted from TSS to TES against the CSA and RefSeq assemblies. The number of hits completely found on a single scaffold is displayed for each assembly. (c) Mouse coding sequences were blasted against Chinese hamster assemblies from the start of translation to the end. (d) The 1,011 complete genes found in PICR were extended 5 kb upstream and 1.5 kb downstream to include promoters and other regulatory noncoding regions and blasted against existing assemblies. (e) Chromatin states around three genes, as found in the previously published CSA‐based chromatin state model (Feichtinger et al., 2016; top for each gene) and the PICR assembly (bottom for each gene), showing promoter and regulatory elements in addition to active transcription. CSA, chromosome‐sorted assembly; TES, transcription end site; TSS, transcription start site [Color figure can be viewed at wileyonlinelibrary.com]

In the previous genome assemblies, many genes were fragmented or separated from their functional genomic elements (e.g., promoters, enhancers, or regions of active or repressed transcription). Thus, efforts to define the chromatin states of genes and their regulatory units were error‐prone (Feichtinger et al., 2016). We therefore recalculated the chromatin states for the PICR assembly using the ChiPSeq‐derived histone mark reads obtained by Feichtinger et al. (2016). In comparison with the previously deduced chromatin states, the emission profile of the new chromatin states matched better with those obtained for the well‐assembled human epigenome (Kundaje et al., 2015; Figure 2a).

To test whether the continuity of genes and their regulatory regions is improved in the PICR meta‐assembly, we extracted a shortlist of 1,538 mitochondria‐associated genes, localized to 1,654 sites in the mouse genome. We mapped the sequences between the mouse transcription start site (TSS) and the transcription end site (TES) against the PICR meta‐assembly, the RefSeq assembly, and the CSA (Brinkrolf et al., 2013; Lewis et al., 2013). Genes were considered present if both the TSS and TES were found on the same scaffold. Due to the high variance in untranslated regions (UTRs) across species, few genes were identified (Figure 2b), demonstrating the importance of a species‐specific genome. We subsequently searched for both the start and the end of the coding sequences on the same scaffold (Figure 2c). Of the complete genes found in PICR (1,011), 85% were annotated and localized to 900 unique locations. The corresponding sequences in PICR were elongated to include UTRs, 5 kb upstream and 1.5 kb downstream, to capture potential regulatory regions, such as promoters or repressive elements. These elongated sequences were mapped against the previously published Chinese hamster genomes (Brinkrolf et al., 2013; Lewis et al., 2013) and again checked for presence on a single scaffold (Figure 2d).

Several genes had their elongated sequence not properly assembled in earlier assemblies, despite having the coding sequence on a single scaffold in each of the three assemblies (Supporting Information Table S6). Examples for three genes, Rab4b, a member of the Ras family of oncogenes, the mitochondrial ribosome protein MRPL27, and TIMM50, a translocase responsible for targeting proteins into the mitochondria, are shown. In all cases, the scaffold in the CSA assembly contained histone marks for active transcription or a genic enhancer, but lacked flanking enhancers and promoter regions. In the new assembly, these are now correctly annotated (Figure 2e). The correct assembly of coding and noncoding regions is of increasing importance to better understand their regulatory function and enable engineering applications. A browser with all PICR scaffolds, the preliminary annotation, and the chromatin states throughout a batch culture is available at http://cgr-referencegenome.boku.ac.at/jb/.

### 3.5 Pervasive gaps are filled by SMRT sequencing

**Figure 3:**
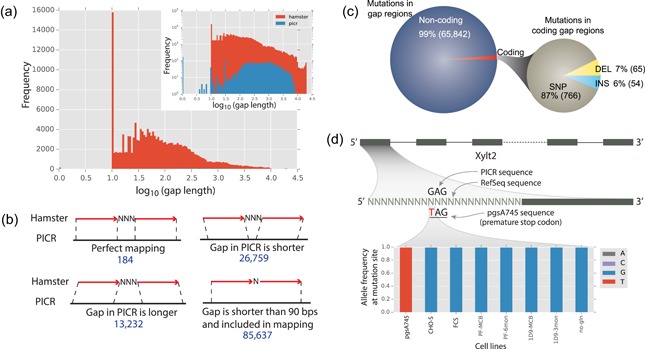
Important variants are located in sequence gaps in previous assemblies. (a) More than 95% of sequence gaps were filled in the PICR meta‐assembly (inset shows the log frequency of gaps to highlight the low frequency of PICR gaps not visible in the normal histogram). (b) The missing sequence in gaps in the RefSeq assembly was identified by aligning the RefSeq sequence flanking the gaps to the PICR sequence. (c) Across 13 cell lines, we found 65,842 SNP and indel mutations in the RefSeq gap regions, and 1.3% of these were found in coding regions. (d) A legacy CHO cell line, pgsA745, identified Xylt2 as the glycosyltransferase responsible for the first step in glycosaminoglycan biosynthesis as this cell line is deficient in glycosaminoglycan biosynthesis. Because of a gap in the RefSeq assembly, only in the new PICR meta‐assembly can the causal variant be identified. A G→T mutation introduces an early stop codon in exon 1, resulting in a loss in Xylt2 activity. The genotype is shown for a variety of CHO cell lines (Feichtinger et al., 2016; Lewis et al., 2013; van Wijk et al., 2017), with only pgsA745 showing the early stop codon. CHO, Chinese hamster ovary; SNP, single nucleotide polymorphism; Xylt2, xylosyltransferase 2 [Color figure can be viewed at wileyonlinelibrary.com]

The RefSeq assembly (Lewis et al., 2013) contains 166,152 gaps with a total length of 58.8 Mb, representing 2.5% of the entire genome. The PICR meta‐assembly has eliminated most gaps, with only 3,238 remaining (Figure 3a). These gaps account for 2.9 Mb, or 0.1%, of the genome. By aligning the RefSeq assembly to PICR using MUMmer3.0 (Kurtz et al., 2004), we identified the missing sequence for 125,812 (76%) of the RefSeq gaps (Figure 3b and the Materials and Methods section). The sequence for a subset of RefSeq gaps was not identified in the PICR meta‐assembly. Of this subset, 90% could not be unambiguously identified because the flanking fragments did not both align to the new assembly, likely due in part to misassemblies in the RefSeq genome (Figure 3).

The elimination of most gaps in the PICR meta‐assembly enables more accurate and complete genome editing and genomic analyses since 2,252 genes in the PICR meta‐assembly had their RefSeq assembly gaps filled. We called variants from whole‐genome resequencing data for 13 representative resequenced CHO cell lines (Feichtinger et al., 2016; Lewis et al., 2013) to identify genes that have newly discovered mutations in the RefSeq coding gaps. Each sample has ∼300 mutations in coding gaps, 90% of which are SNPs (Supporting Information Table S7). Across 13 cell lines, 885 novel variants in coding gaps were found in 134 genes (Figure 3c).

Gene classes with the highest gap filling success included genes associated with protein binding, RNA binding, and transcription (Supporting Information Figure S6), including genes containing zinc finger motifs and ribosomal genes. Previously, such genes were replete with gaps due to their conserved domains shared across many other genes in the genome. We further explored which genes had coding mutations in their filled gaps. The top GO terms for these 225 genes are also enriched in DNA binding and transcription (Supporting Information Figure S7). In summary, the gaps in the previous assembly could potentially confound genomic studies in CHO, especially those involving mutations associated with DNA or RNA binding, including transcription factors.

#### 3.5.1 An important mutation in Xylt2 is found within a filled sequence gap

Beyond their importance in biopharmaceutical production, CHO cells were fundamental to cell biology and biochemistry research for many decades. For example, genetic screens of many CHO cell lines were used to identify glycosyltransferases (Maeda, Ashida, & Kinoshita, 2006; Patnaik & Stanley, 2006; Stanley, 2014; Zhang, Lawrence, Frazier, & Esko, 2006) and genetic mapping efforts were deployed to identify causal mutations. The pgsA745 cell line (van Wijk et al., 2017) has been used for decades in the glycobiology field due to its deficiencies in glycosaminoglycan synthesis (Esko, Stewart, & Taylor, 1985), due to a truncation of the Xylt2 protein (Cuellar, Chuong, Hubbell, & Hinsdale, 2007). However, upon variant calling from whole‐genome resequencing data for the pgsA745 cell line (van Wijk et al., 2017) using the RefSeq assembly, we failed to identify the causal mutation, whereas a G‐>T SNP encoding a premature stop codon was found in exon 1 of Xylt2 when using the PICR genome assembly (Figure 3d). This mutation was previously missed since the RefSeq assembly has a gap of 447 bp that spanned the first exon on scaffold NW_003613846.1. However, this gap was filled in PICR, enabling the identification of the mutation. Thus, filling of the gap sequence leads to a valuable improvement to genomic studies, including the identification of causal variants in CHO cell lines.

## 4 DISCUSSION

For 60 years, CHO cells have been invaluable for biomedical research and fundamental to the study of several biological processes, such as glycosylation (Goh et al., 2014) and DNA repair (Thompson et al., 1987). In addition, for >30 years, they have been the host cell of choice for the production of most biotherapeutics. Although the aforementioned research was carried out without genomic resources, new opportunities are arising with published CHO genome sequences (Brinkrolf et al., 2013; Lewis et al., 2013; Xu et al., 2011; Yusufi et al., 2017). However, the draft nature of these genome sequences poses challenges for many applications. Here, we present a major step forward in further facilitating the adoption of cutting‐edge technologies for cell line development and engineering.

The primary outcome here is a substantially improved reference genome sequence for the Chinese hamster. Specifically, the N50 of the PICR meta‐assembly is 13× the length of the RefSeq assembly N50, and we reduced the number of scaffolds to 1/29 the number in RefSeq. Furthermore, we demonstrated that the initial PICR assembly only had one detected misassembly, whereas the RefSeq assembly had at least 24 > 1 Mb scaffolds with cross‐chromosome misassemblies (Supplementary Figure S2). Finally, we eliminated more than 95% of the gap sequence in the current RefSeq assembly, and provide a more complete and contiguous view of the genomic sequence of the Chinese hamster.

Various aspects of the genome assembly were improved by merging the different datasets and data types. First, merging the Illumina reads from two different genome sequencing efforts resulted in a higher quality genome than the starting assemblies. Second, further improvements in the assembly attributes were achieved by merging the single‐platform assemblies. Previously, assembly merging with Metassembler was found to modestly improve the starting assemblies (Bradnam et al., 2013). Here, we obtained large gains in the N50, with the PICR meta‐assembly being ∼4× more contiguous than the starting assemblies. Medium and longer scaffolds were successfully merged, thus reducing the number of N50 and N90 scaffolds. However, by including Illumina‐based assemblies, many short scaffolds remained, as seen in the lower median scaffold length in the PICR meta‐assembly compared with the curated PacBio SMRT assembly. The merged assembly thus benefited both from the longer reads from the PacBio SMRT contigs and the longer scaffolds from the large insert size libraries used for the Illumina assemblies. It is anticipated that the use of optical mapping and chromatin interaction mapping (Bickhart et al., 2017) would further extend the scaffolds and span large repeat regions, resulting in more complete chromosomal maps for the Chinese hamster.

Despite the absence of genomic resources, CHO‐based bioprocessing has advanced substantially for ∼30 years. Massive improvements in protein titer were predominantly achieved through media and process optimization. Systematic optimization of CHO cell lines itself has lagged behind *Escherichia coli* and *Pichia pastoris* and has only recovered traction with the comparatively late release of draft genomes. The availability of genomic data now enables improved control over product quality and more predictable culture phenotypes. For example, more contiguous and complete sequences will facilitate the identification of sites for targeted integration of transgenes, enabling more reproducible productivity across clones (Lee, Kallehauge, Pedersen, & Kildegaard, 2015) and reducing the burden of stability testing. In addition, the elimination of gap sequence regions enables the improved identification of genomic variants and design of genome editing tools. Furthermore, by sequencing through repetitive elements, endogenous retroviral elements can be deleted. This could substantially reduce the retroviral particles secreted in mammalian cell culture (Anderson, Low, Lie, Keller, & Dinowitz, 1991; Wheatley, 1974), increase biopharmaceutical safety, and decrease the burden of adventitious agent testing and purification. Comparable efforts have successfully cleaned up similar elements in the porcine genome (Yang et al., 2015).

The full benefit of this more contiguous genome will become apparent as novel genome‐editing tools are applied to control cell phenotypes. These include efforts to delete larger tracts of the sequence, including genes, promoters, and other regulatory elements using paired gRNAs that remove the entire sequence rather than only introducing frameshifts (Schmieder et al., 2017). Thus, genes can be removed or promoters can be replaced with synthetic or inducible elements. Furthermore, with more complete regulatory element sequences, one could use CRISPRa/i to regulate gene expression levels. Finally, tools can be deployed that modify the methylation of endogenous promoters to activate or silence gene expression (Morita et al., 2016; Vojta et al., 2016). Overall, these strategies enhance our control over cell phenotype. As demonstrated, these precision engineering tools are highly dependent on the availability of a contiguous and well‐assembled genome, as presented here, to the entire scientific and industrial community.

## AUTHOR CONTRIBUTIONS

O.R., S.G., and K.B. conducted genome assembly, and contributed toward writing. M.L.M. functionally annotated the genome, and contributed toward writing. H.D. and I.H. performed the mitochondrial gene and chromatin state analysis. S.L. conducted gap analysis, prepared CHO pgsA‐745 DNA for sequencing, and contributed toward writing. K.H. isolated hamster tissue and prepared DNA for sequencing. M.J.B. conceived of the project and oversaw the hamster DNA preparation. S.P. provided valuable guidance and contributed toward the sequencing and analysis. H.H., B.K., and M.S. contributed toward the sequencing and analysis. V.J. evaluated approaches to separate scaffolds for chromosomes 9 and 10. A.G. oversaw genome assembly efforts. N.E.L. conceived of the project, wrote the manuscript, and guided the gap analysis. N.B. conceived of and coordinated the project, oversaw the chromatin state analysis and wrote the manuscript. K.H.L. conceived of and coordinated the project, and oversaw the genome annotation.

## Supporting information

Supporting information

Supporting information

Supporting information

Supporting information
