# Supplementary material for: A reference genome of the Chinese hamster based on a hybrid assembly strategy
Source: Biotechnol Bioeng. 2018 May 29;115(8):2087–100. doi: 10.1002/bit.26722 (PMC6045439; doi:10.1002/bit.26722)
Supplement: Supplementary file 4 — Supporting information [file BIT-115-2087-s004.docx]

**Supplementary Material**

**Supplementary Figures**


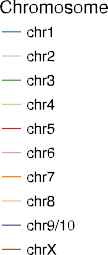


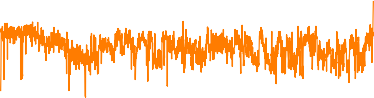


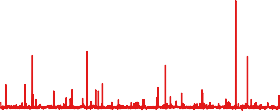


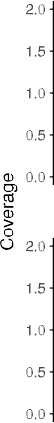


Figure S1: Normalized coverage plots identify misassembly sites. Chinese hamster chromosomes were previously sorted and sequenced separately [Brinkrolf et al., 2013], and reads were aligned to each scaffold. Each color indicates aligned reads from different chromosomes. The top image shows a scaffold in which the normalized coverage shows all reads were from a single chromosome. The bottom plot shows a clear assembly error in which the first 10 Mb are covered by a different chromosome (pink) than the remaining part (blue).


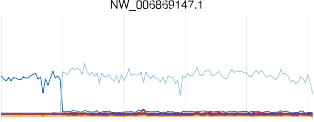


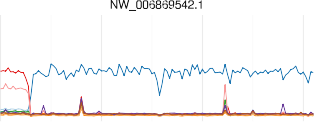


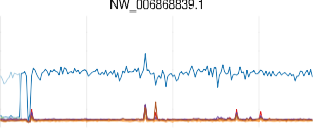


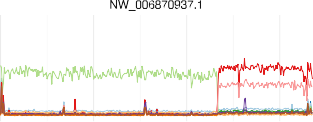


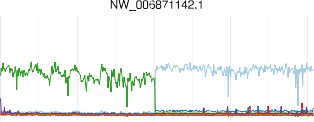


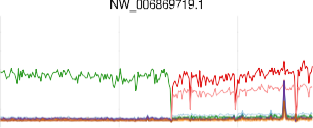


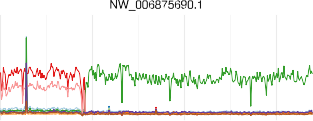


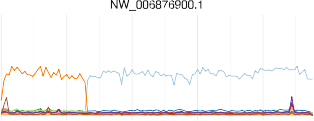


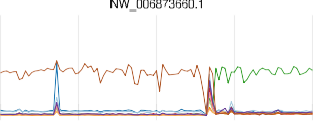


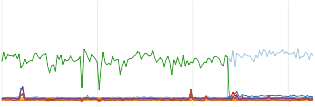


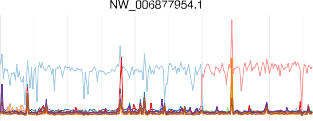


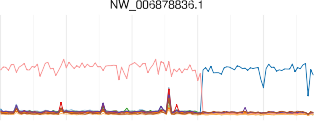


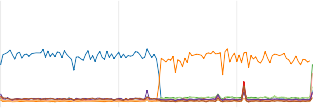


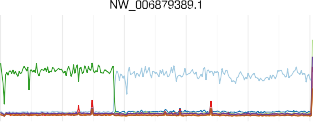


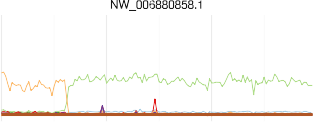


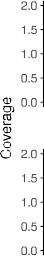


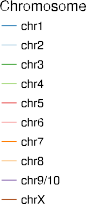


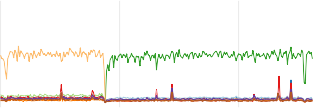


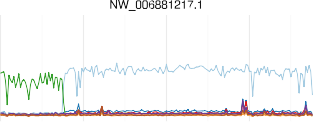


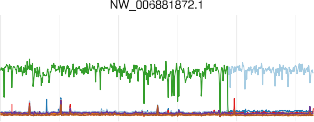


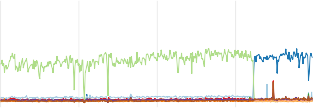


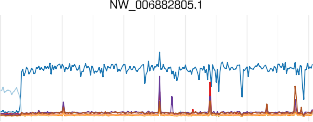


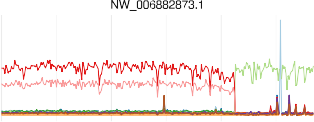


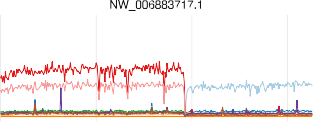


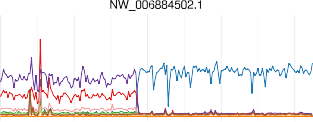


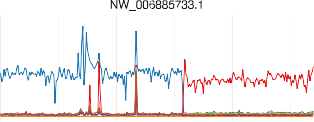


Figure S2: Chromosome-separated reads were realigned to all scaffolds larger than 1 Mb in the RefSeq assembly. From these, 24 scaffolds were identified that had more than 5% of the scaffold not associated with the primary chromosome.


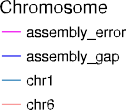


Figure S3: Zoomed chromosome coverage plot at the assembly error region of PICR scaffold 7. A 30 kb region (pink bar) with low and mixed coverage and assembly gap (blue bars) is clearly visible.


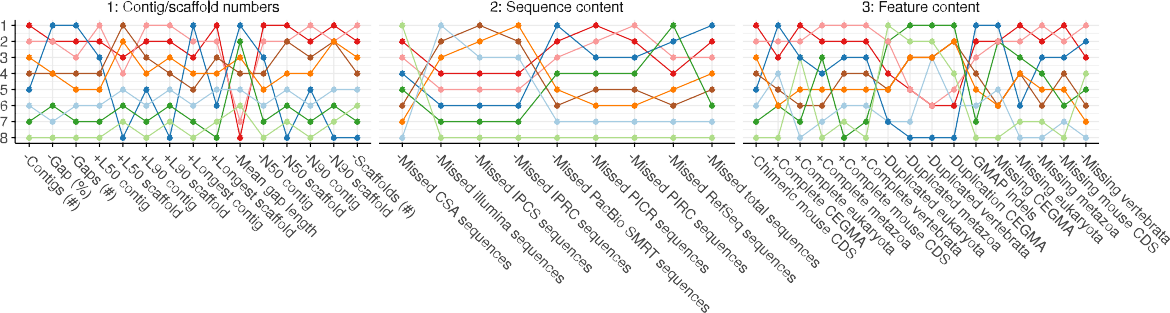


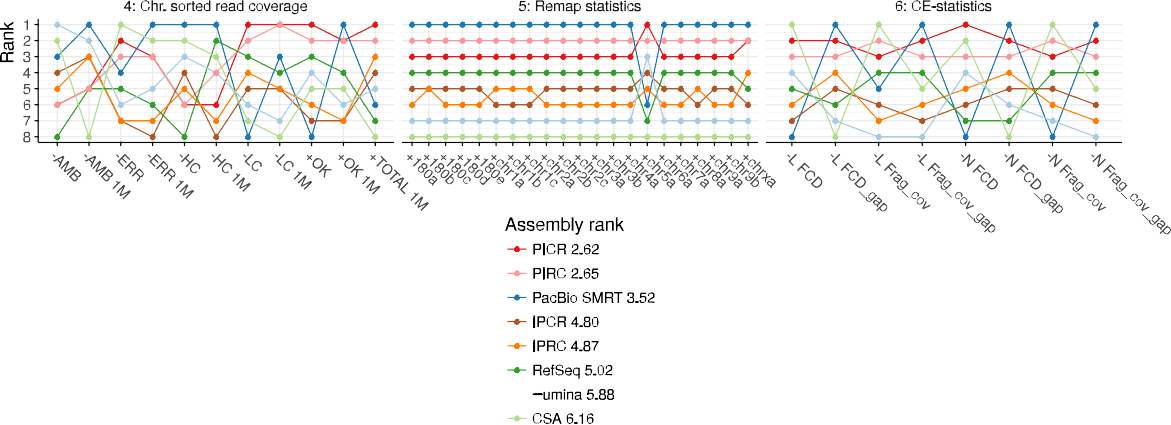


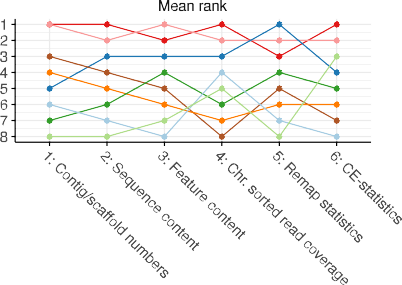


Figure S4: Ranks of the assemblies for all metrics in all classes.


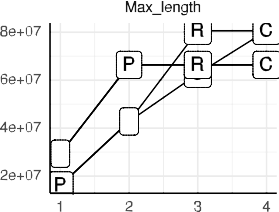

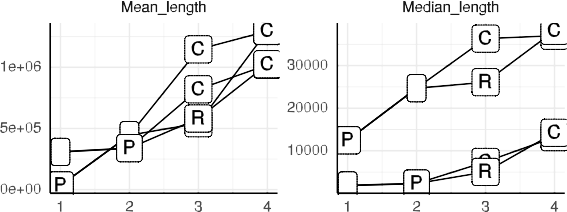

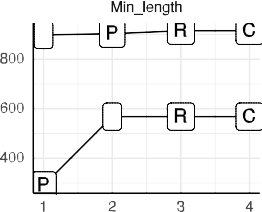


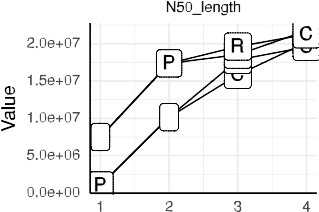

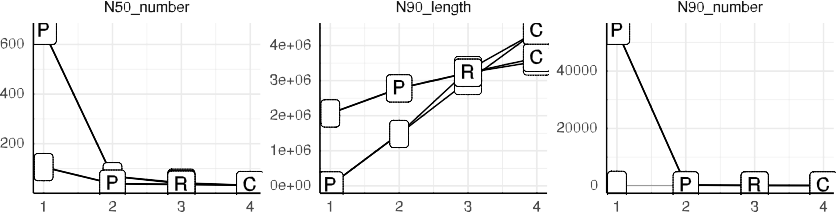


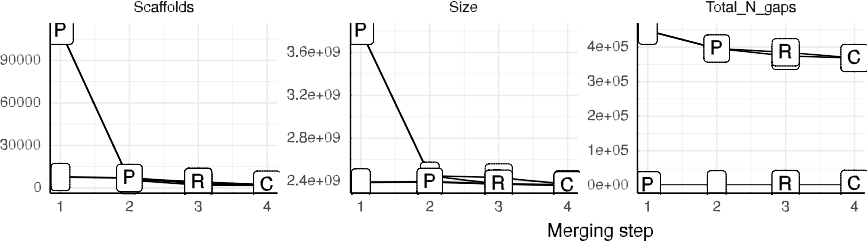

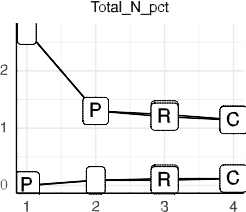


Figure S5: Assembly metrics at the different stages of the metassemblies. Improvement in all stages of the metassembly can be seen in most of the metrics.


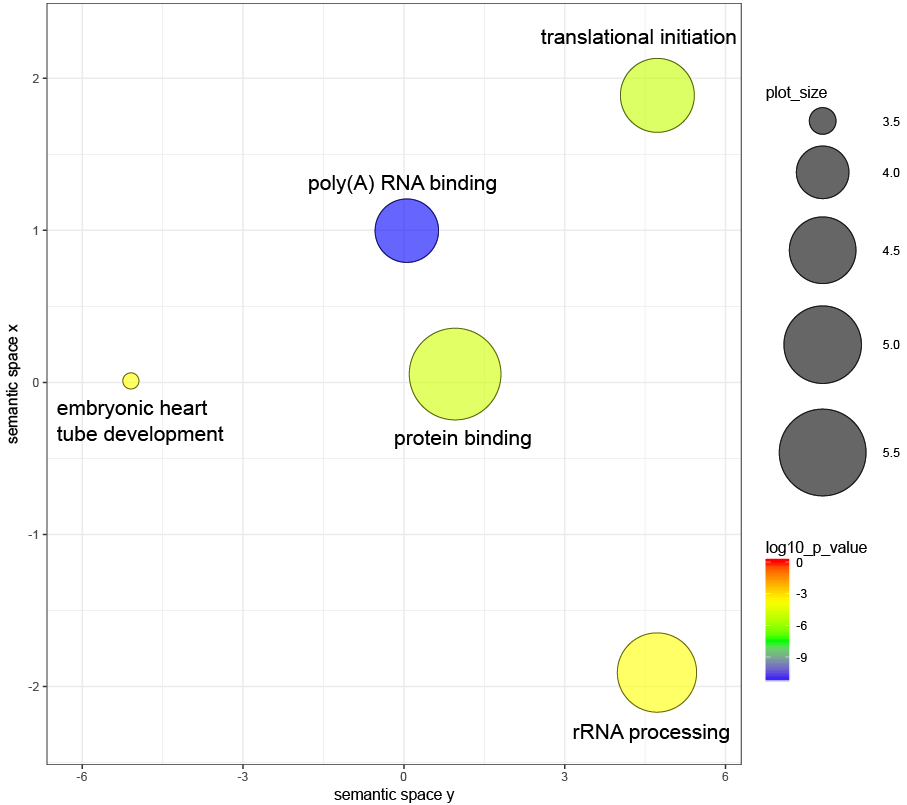


Figure S6: GO term analysis of genes with coding gaps. Enriched GO terms were identified using DAVID. 2,252 genes with coding gaps were searched against the whole human GO term sets. The top 5 GO terms with FDR corrected p-value smaller than 0.05 were shown in the figure using REVIGO. Circle size represents the relative gene set size of each GO term compared to the whole human gene sets. This indicates genes related to transcription or translational regulation may be difficult to fully assemble only using Illumina reads, as some classes of transcription factors and other oligonucleotide binding proteins have highly conserved or repetitive sequences (e.g., zinc finger proteins).


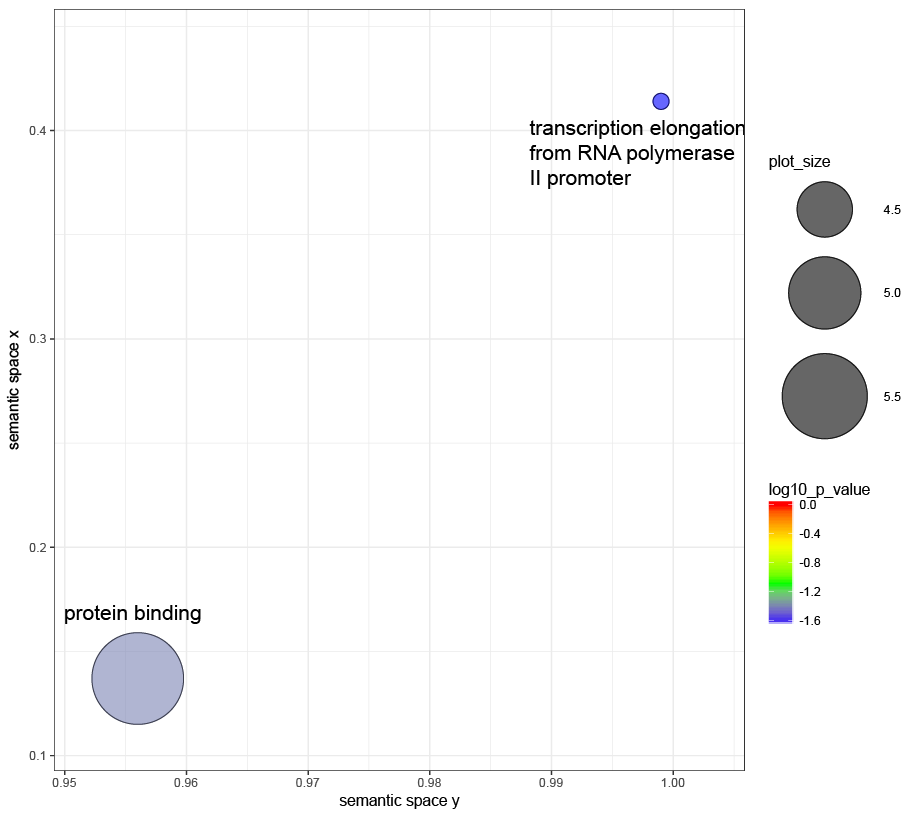


Figure S7: GO term analysis of genes with mutations in their coding gaps. Enriched GO terms were identified using DAVID. 134 genes with variants in their coding gaps were searched against all of the 2,252 genes with coding gaps. GO terms with an FDR-corrected p-value smaller than 0.05 were shown in the figure using REVIGO. Circle size represents the relative gene set size of each GO term compared to the whole human gene sets. This indicates genes related to transcription regulation tend to have more mutations than the other genes with coding gaps.


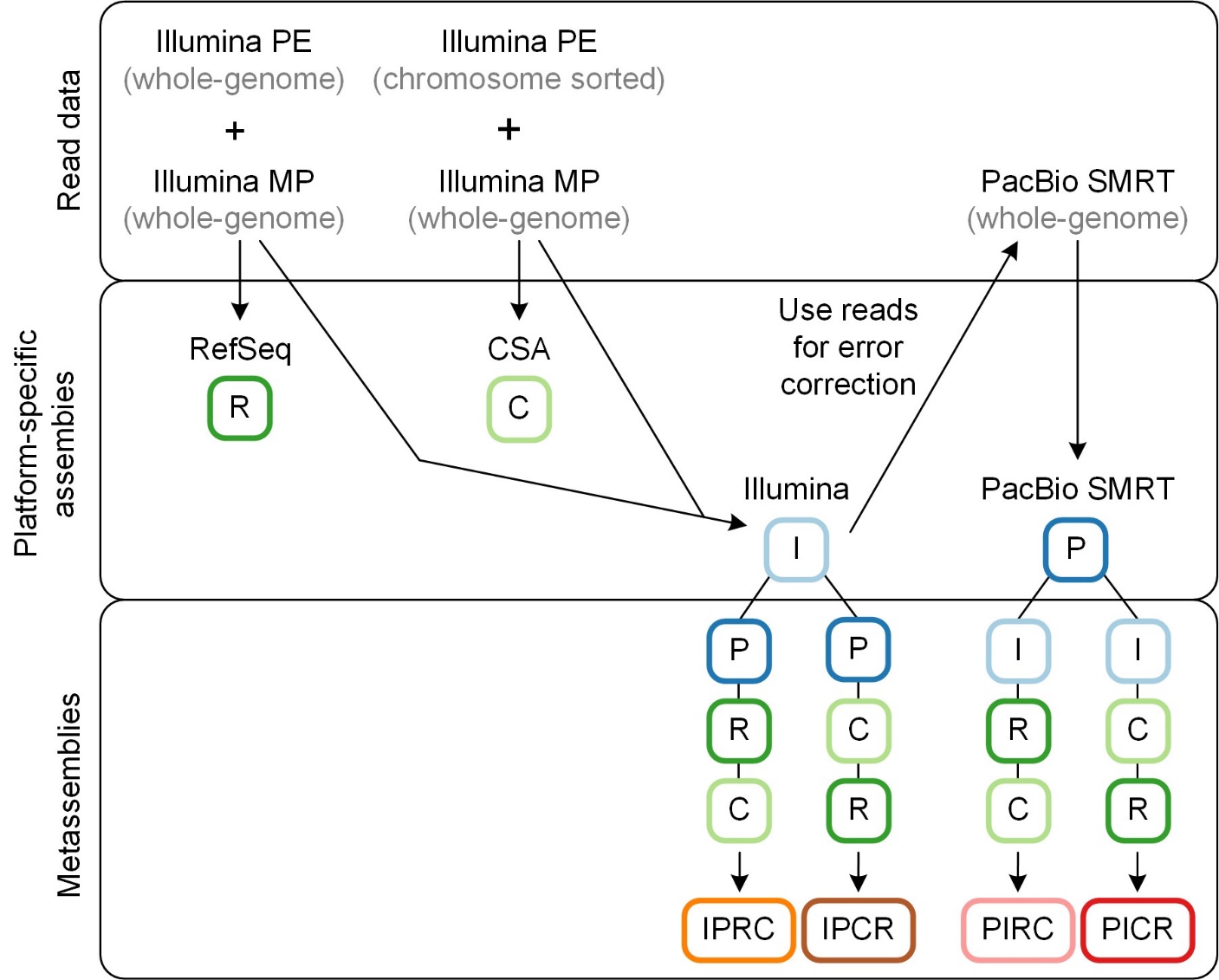


Figure S8: Overview of the complete assembly workflow. First, two primary assemblies were built, using all Illumina reads (I) or Pacific Biosciences SMRT reads (P). In addition, previously published assemblies were collected, which were based on chromosome separated reads (C) or the whole genome RefSeq (R). The four assemblies were then iteratively merged in four different orders, creating four metassemblies (IPCR, IPRC, PICR, PIRC). The best assembly of all eight assemblies was chosen based on a panel of 80 metrics.


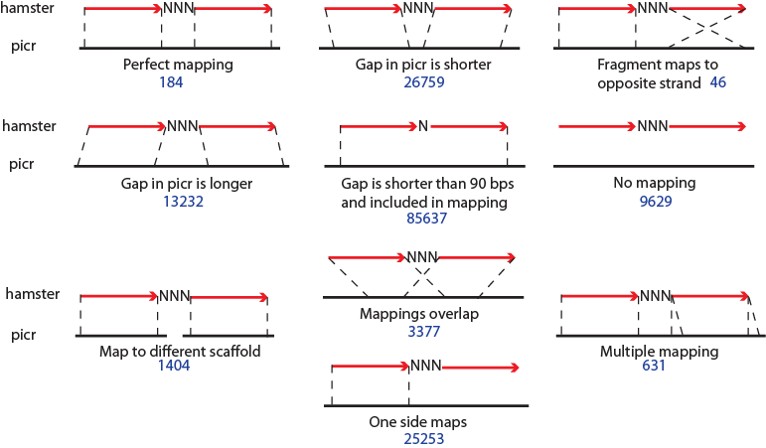


Figure S9: Matches around and including gaps were used to identify the corresponding region in PICR genome. Numbers in the figure represent the amount of the specific gaps we identified.


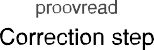


Figure S10: Distribution of the indel-ratios (number of indel per matched base) of the raw (red) and error-corrected PacBio reads (green: indel-ratio after first correction step, blue: final indel-ratio).


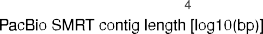


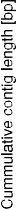


Figure S11: Weighted contig length histogram of the PacBio SMRT metassembly shows a clear bimodal distribution (logarithmic length of the contigs is on the x-axis, sum of the length of all contigs per bin on the y-axis). The total size of the second mode is close to the estimated genome size. About 73% of the second mode contigs (ca. 66% in size) can be completely aligned to the contigs from the first mode.


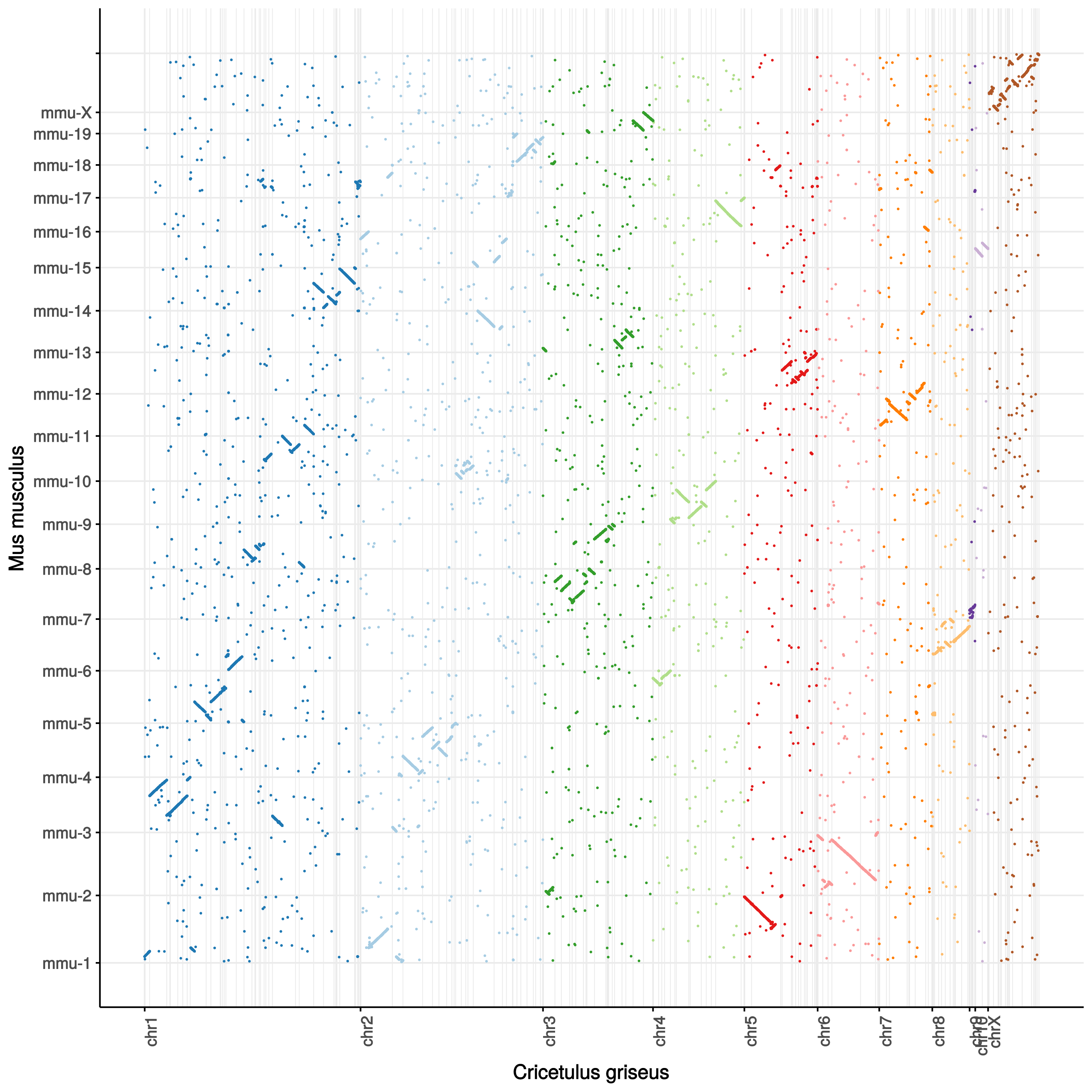


Figure S12. The dotplot visualizes the NUCmer alignments of the PICR scaffolds to the mouse chromosomes. The PICR scaffolds are ordered by chromosome and position of the longest alignment to the mouse genome.


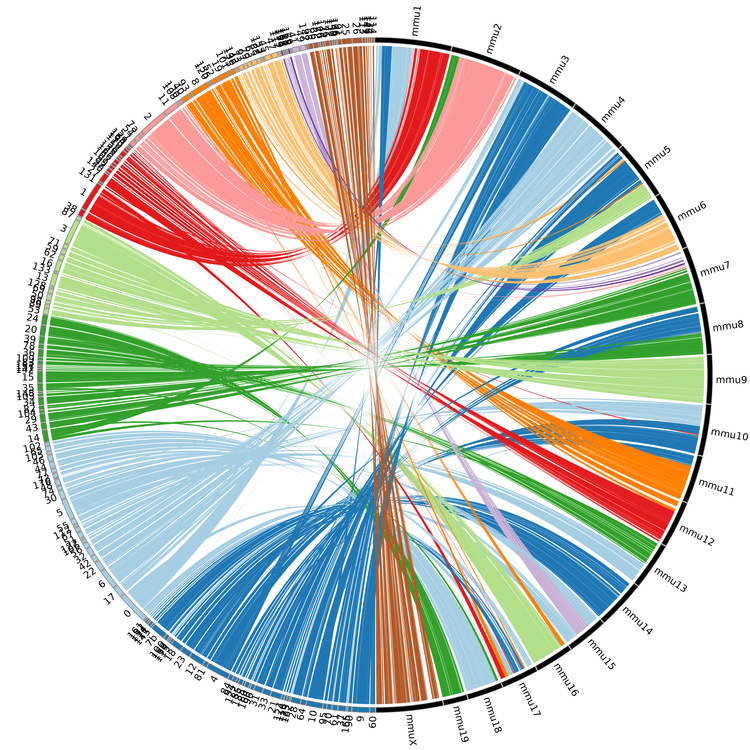


Figure S13. The alignment chains produced by aligning the PICR scaffolds to the mouse chromosomes using NUCmer are shown in the circular plot. The mouse chromosomes on the right side are shown in black and the PICR scaffolds on the left are ordered and colored by hamster chromosome.


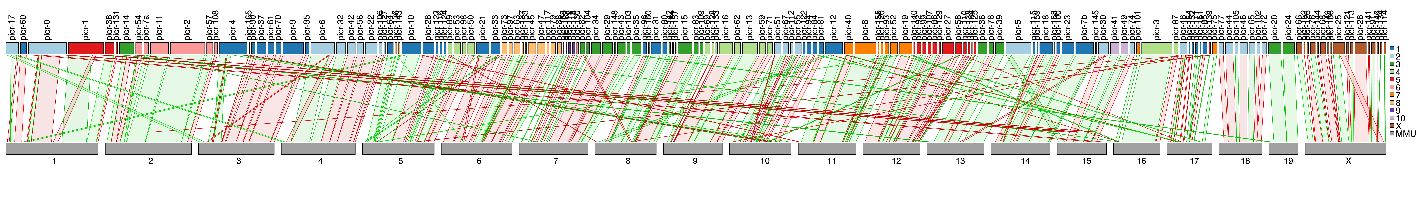


Figure S14. The alignment chains produced by aligning the PICR scaffolds to the mouse chromosomes using NUCmer are shown in the linear plot.


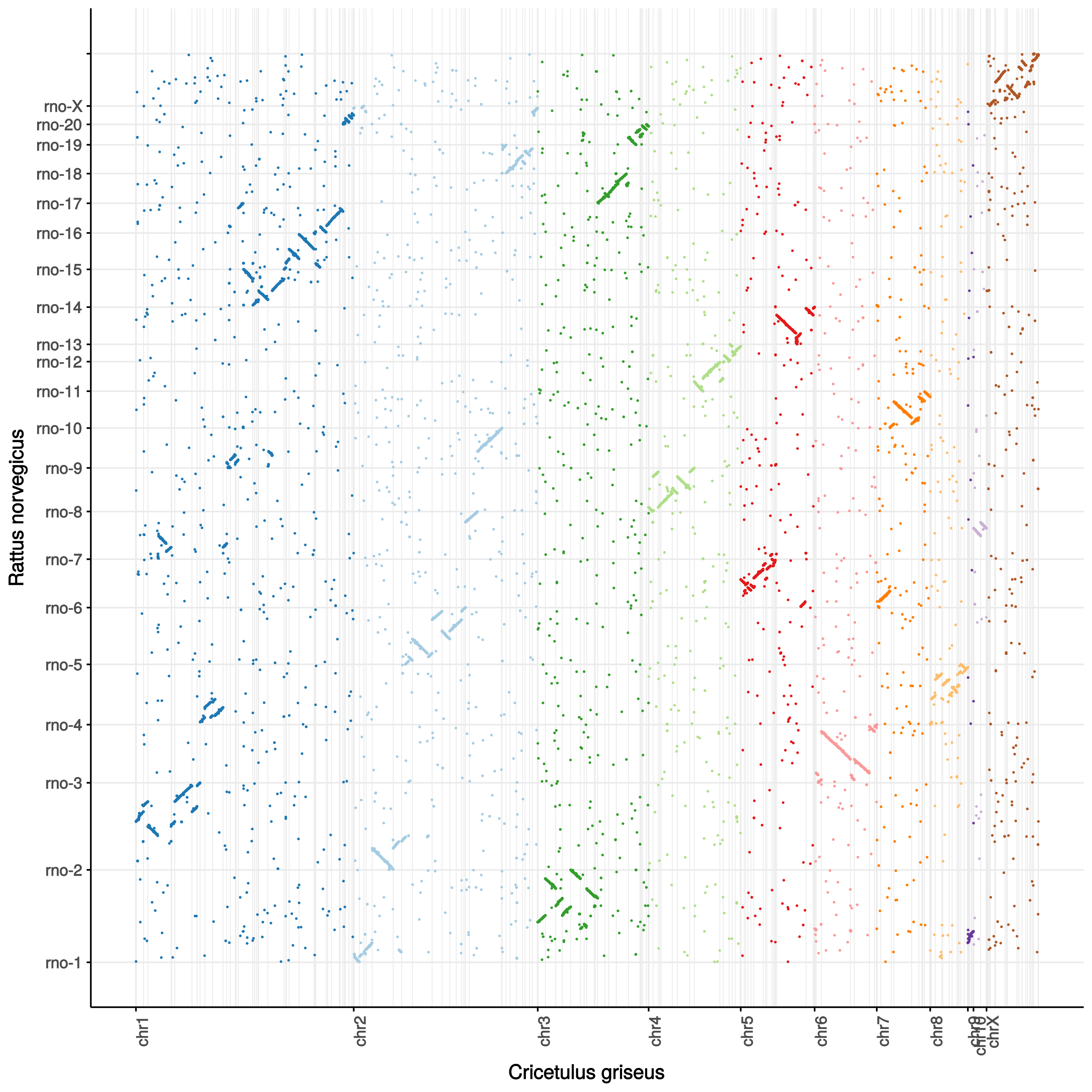


Figure S15. The dotplot visualizes the NUCmer alignments of the PICR scaffolds to the rat chromosomes. The PICR scaffolds are ordered by chromosome and position of the longest alignment to the mouse genome.


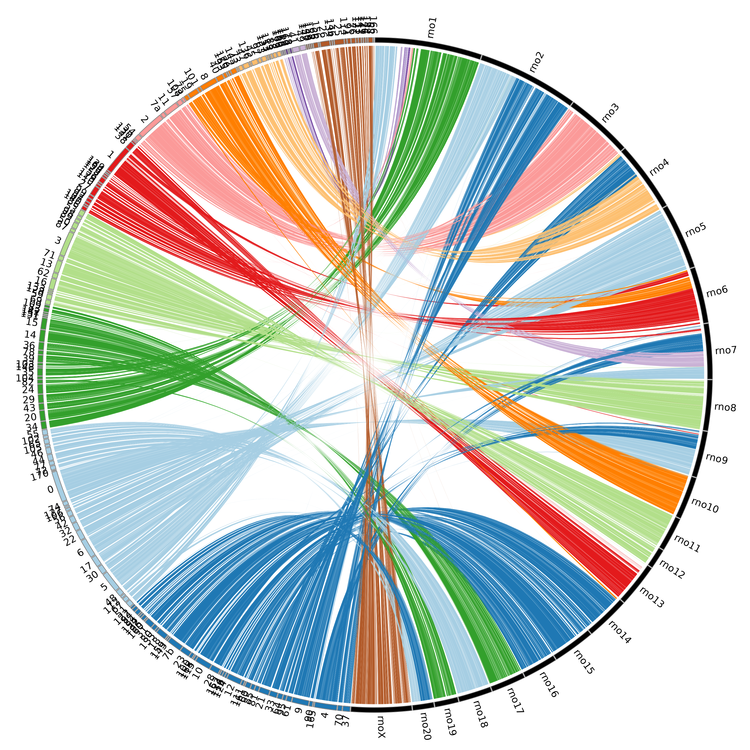


Figure S16. The alignment chains produced by aligning the PICR scaffolds to the rat chromosomes using NUCmer are shown in the circular plot. The rat chromosomes on the right side are shown in black and the PICR scaffolds on the left are ordered and colored by hamster chromosome.


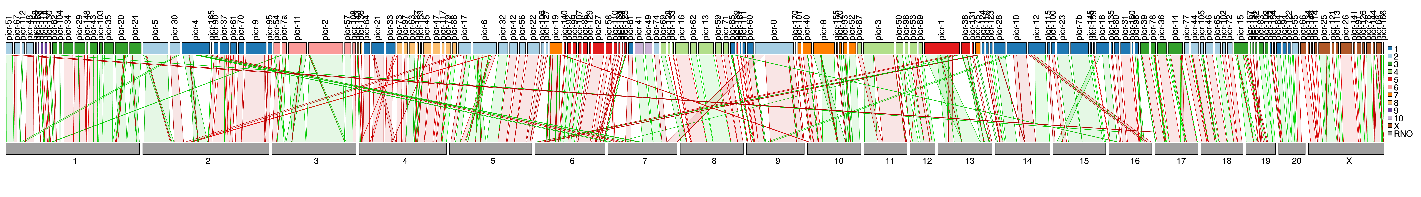


Figure S17. The alignment chains produced by aligning the PICR scaffolds to the rat chromosomes using NUCmer are shown in the linear plot.

**Supplementary Tables**

|  | PacBio SMRT  metassembly | Mode-2  (contigs *≥* 100 kb) | Mode-1  (contigs < 100 kb) |
| --- | --- | --- | --- |
| Contigs [#] | 110,954 | 1,659 | 109,295 |
| Length [Gb] | 3.8 | 2.31 | 1.49 |
| Min length [bp] | 290 | 100,560 | 290 |
| Max length [Mb] | 16.08 | 16.08 | 0.09 |
| Mean length [kb] | 34.28 | 1,394.69 | 13.63 |
| Median length [bp] | 12,460 | 693,156 | 12,285 |
| N50 length [kb] | 995.27 | 2,906.73 | 18.52 |
| N50 [#] | 655 | 223 | 28,878 |
| N90 length [kb] | 12.76 | 623.9 | 8.07 |
| N90 [#] | 54,054 | 884 | 74,583 |

Table S1: Assembly metrics of the complete PacBio SMRT metassembly and the contigs larger 100 kb or smaller 100 kb.

| Chromosome | Number of scaffolds | Bases [Mb] |
| --- | --- | --- |
| 1 | 73 | 549.76 |
| 2 | 35 | 463.59 |
| 3 | 42 | 281.86 |
| 4 | 24 | 231.54 |
| 5 | 56 | 188.78 |
| 6 | 9 | 155.90 |
| 7 | 13 | 134.49 |
| 8 | 24 | 96.66 |
| 9 | 15 | 18.79 |
| 10 | 2 | 32.58 |
| X | 48 | 134.88 |
| unassigned | 1,489 | 80.08 |

Table S2: Number and size of scaffolds assigned to each chromosome

Table S3: See Table_S3_metric_description.xls

| Assembly | | |
| --- | --- | --- |
| All genes | PICR | IPCR |
| Gene count | 24,686 | 23,410 |
| Transcript count | 24,948 | 23,656 |
| Transcripts per gene | 1.01 | 1.01 |
| Avg. length transcript | 17,615.04 | 18,089.17 |
| Total length transcript | 439,460,104 | 427,917,413 |
| Avg. coding length | 1,324.93 | 1,316.11 |
| Total coding length | 33,054,355 | 31,133,905 |
| Avg. exons per transcript | 7.49 | 7.54 |
| Total exons | 186,939 | 178,277 |
| Complete transcripts | PICR | IPCR |
| Transcript count | 18,476 | 17,557 |
| Avg. length transcript | 18,908.94 | 19,434.05 |
| Med. length transcript | 8,236 | 8,228 |
| Total length transcript | 349,361,499 | 341,203,668 |
| Avg. coding length | 1,334.19 | 1,317.74 |
| Med. coding length | 981 | 966 |
| Total coding length | 24,650,545 | 23,135,510 |
| Avg. exons per transcript | 7.49 | 7.48 |
| Total exons | 138,358 | 131,262 |
| Incomplete transcripts | PICR | IPCR |
| Transcript count | 6,472 | 6,099 |
| Avg. length transcript | 13,921.29 | 14,217.70 |
| Med. length transcript | 8,128 | 8,692 |
| Total length transcript | 90,098,605 | 86,713,745 |
| Avg. coding length | 1,298.49 | 1,311.43 |
| Med. coding length | 933 | 942 |
| Total coding length | 8,403,810 | 7,998,395 |
| Avg. exons per transcript | 7.51 | 7.71 |
| Total exons | 48,581 | 47,015 |

Table S4: Gene and transcript information from the Maker annotation of the PICR and IPCR genome assemblies.

| Repeat class | PICR | IPCR |
| --- | --- | --- |
| Simple repeats | 2,237,638 | 2,516,964 |
| Low complexity repeats | 271,488 | 274,248 |
| Long terminal repeats | 625,480 | 601,600 |
| LINEs | 882,602 | 858,268 |
| SINEs | 1,282,452 | 1,227,136 |
| Satellites | 8,436 | 14,714 |
| Retro-transposons | 3,998 | 4,422 |
| DNA repeat elements | 146,430 | 170,094 |
| RNA repeats | 4,940 | 4,828 |
| Other | 23,522 | 22,470 |
| Unknown | 48,586 | 44,368 |
| Total masked | 5,535,572 | 5,739,112 |

Table S5: Number of repeats by class masked in PICR and IPCR assemblies prior to annotation.

Table S6: See Table_S6_supplementary_mito.xlsx

|  | pgsa | CHOS | FCS | PF-MCB | PF_6mon | 1D9-MCB | 1D9_3mon | no_gln | C0101 | CHOS | CHOpr | DG44 | ECACC | K1_SF |
| --- | --- | --- | --- | --- | --- | --- | --- | --- | --- | --- | --- | --- | --- | --- |
| Total variants | 5,693,728 | 4,663,123 | 5,948,137 | 5,813,705 | 5,548,170 | 5,901,401 | 5,894,448 | 5,833,668 | 5,566,074 | 4,663,123 | 5,042,526 | 4,727,716 | 5,025,295 | 5,049,364 |
| Control variants | 1,052,383 | 861,231 | 1,122,569 | 1,072,086 | 993,010 | 1,133,805 | 1,138,751 | 1,061,842 | 1,050,577 | 861,231 | 896,154 | 852,616 | 879,678 | 889,065 |
| Control ftltered  variants | 4,641,345 | 3,801,892 | 4,825,568 | 4,741,619 | 4,555,160 | 4,767,596 | 4,755,697 | 4,771,826 | 4,515,497 | 3,801,892 | 4,146,372 | 3,875,100 | 4,145,617 | 4,160,299 |
| Filtered snp | 3,764,166 | 3,120,614 | 3,849,415 | 3,758,389 | 3,612,053 | 3,727,231 | 3,725,799 | 3,781,099 | 3,629,854 | 3,120,614 | 3,394,811 | 3,190,921 | 3,383,881 | 3409772 |
| Filtered ins | 426,252 | 327,492 | 481,742 | 480,580 | 467,250 | 518,096 | 511,587 | 491,032 | 428,353 | 327,492 | 363,120 | 326,771 | 369,174 | 361,985 |
| Filtered del | 450,927 | 353,786 | 494,411 | 502,650 | 475,857 | 522,269 | 518,311 | 499,695 | 457,290 | 353,786 | 388,441 | 357,408 | 392,562 | 388,542 |
| Gap variants | 20,635 | 13,997 | 25,724 | 25,204 | 24,327 | 27,810 | 27,123 | 26,304 | 20,417 | 13,997 | 15,434 | 14,035 | 15,563 | 15,586 |
| Gap snp | 15,924 | 11,327 | 18,778 | 17,797 | 17,404 | 19,386 | 19,052 | 18,773 | 15,623 | 11,327 | 12,285 | 11,389 | 12,210 | 12,430 |
| Gap ins | 1,788 | 999 | 3,201 | 3,407 | 3,175 | 3,969 | 3,684 | 3,506 | 1,828 | 999 | 1,158 | 987 | 1,220 | 1,141 |
| Gap del | 2,923 | 1,671 | 3,745 | 4,000 | 3,748 | 4,455 | 4,387 | 4,025 | 2,966 | 1,671 | 1,991 | 1,659 | 2,133 | 2,015 |
| Coding gap variants | 268 | 212 | 330 | 333 | 294 | 348 | 339 | 366 | 277 | 212 | 207 | 178 | 195 | 200 |
| Coding gap snp | 249 | 199 | 293 | 285 | 274 | 303 | 290 | 310 | 259 | 199 | 195 | 168 | 186 | 188 |
| Coding gap ins | 6 | 3 | 16 | 25 | 6 | 20 | 23 | 28 | 6 | 3 | 3 | 3 | 4 | 2 |
| Coding gap del | 13 | 10 | 21 | 23 | 14 | 25 | 26 | 28 | 12 | 10 | 9 | 7 | 5 | 10 |

Table S7: Variant statistics in different CHO cell lines.

| RefSeq assembly [Lewis et al., 2013] | | | |
| --- | --- | --- | --- |
| Insert size | Bases (Gb) | Number of read-pairs | Mean read length |
| 180 | 92.62 | 497,911,030 | 93.00 |
| 500 | 61.52 | 330,723,818 | 93.00 |
| 800 | 26.64 | 143,168,236 | 93.00 |
| 2000 | 30.34 | 188,590,215 | 80.55 |
| 5000 | 14.64 | 88,225,147 | 83.00 |
| 10000 | 12.48 | 75,094,089 | 83.00 |
| 20000 | 2.42 | 15,063,393 | 80.00 |
| CSA assembly [Brinkrolf et al., 2013] | | | |
| Insert size / chromosome | Bases (Gb) | Number of read-pairs | Mean read length |
| 180 / chr1 | 34.30 | 111,436,801 | 153.97 |
| 180 / chr2 | 33.10 | 107,485,431 | 154.06 |
| 180 / chr3 | 19.96 | 65,249,415 | 153.03 |
| 180 / chr4 | 10.34 | 34,434,540 | 150.00 |
| 180 / chr5 | 9.26 | 30,834,375 | 150.00 |
| 180 / chr6 | 10.66 | 35,519,916 | 150.00 |
| 180 / chr7 | 5.22 | 20,881,069 | 125.00 |
| 180 / chr8 | 7.62 | 30,451,947 | 125.00 |
| 180 / chr9/10 | 18.74 | 61,114,204 | 153.37 |
| 180 / chrx | 11.06 | 35,424,777 | 156.00 |
| 4,500 | 10.48 | 51,509,899 | 101.73 |
| 6,000 | 16.30 | 63,722,909 | 132.70 |
| 6,200 | 14.52 | 71,488,589 | 101.50 |

Table S8: Overview of the different Illumina libraries.

|  | PICR contigs | Mouse contigs | Rat contigs | RefSeq contigs |
| --- | --- | --- | --- | --- |
| Contigs [#] | 4,517 | 445 | 32,025 | 117,912 |
| Length [Gb] | 2.37 | 2.65 | 2.65 | 2,31 |
| Min length [bp] | 104 | 562 | 84 | 201 |
| Max length [Mb] | 14.60 | 91.93 | 2.18 | 0.76 |
| Mean length [kb] | 523.81 | 5,949.87 | 82.85 | 19.55 |
| Median length [bp] | 52,173 | 26,251 | 36,272 | 1,134 |
| N50 length [kb] | 2,446.66 | 32,813.18 | 200.97 | 84.26 |
| N50 [#] | 270 | 25 | 3,665 | 8,297 |
| N90 length [kb] | 418.89 | 8,381.66 | 44.49 | 20.86 |
| N90 [#] | 1,148 | 82 | 14,494 | 28,528 |

Table S9: Contig size metrics for the PICR assembly compared to Ensembl mouse and rat chromosome contigs and RefSeq hamster contigs. For all assemblies, chromosomes and scaffolds were split at all ‘N’-stretches at least 100 bp long to enable consistent comparison between assemblies.

| Assembly | | | |
| --- | --- | --- | --- |
| Gene prediction | RefSeq  (GCF_000419375.1) | PICR | IPCR |
| Total genes Protein-coding Partial  Total CDS Corrected CDS  have premature stops have frameshifts  Transcripts with no support  Transcript with partial support | 27,982  20,678  2,576  32,329*  2,039  747  1,583  105  5,710 | 26,931  20,668  526  27,205  963  332  752  217  4,192 | 25,802  20,074  516  25,884  1,021  521  670  173  4,763 |

Table S10: NCBI annotation of the RefSeq (C_griseus_v1.0), PICR, and IPCR assemblies. *The larger number of CDSs for C_griseus_v1.0 is a result of the pipeline rules for making alternative variants, which allow more variants for the assembly that NCBI considers the reference (currently C_griseus_v1.0). Because NCBI does not create alternative variants for genes that are corrected, this rule does not impact the direct comparison of the corrected CDSs.

| Assembly | | | |
| --- | --- | --- | --- |
| Mouse transcript alignments | RefSeq  (GCF_000419375.1) | PICR | IPCR |
| Number of coding RefSeq  transcripts | 30,782 | 30,782 | 30,782 |
| Aligned | 29,146 | 29,131 | 28,832 |
| Unaligned | 1,636 | 1,651 | 1,948 |
| Split alignment | 1,479 | 424 | 486 |
| Corrected CDS | 2,039 | 963 | 1,021 |
| <95% CDS coverage | 10,445 | 5,342 | 6,655 |

Table S11: NCBI alignment of mouse coding transcripts from RefSeq (NM_prefix) to the RefSeq C_griseus_v1.0, PICR, and IPCR genome assemblies.

Table S12: See Table_S12_PIRC_to_PICR_alignment.xlsx

# Additional information on SMRT sequencing and assembly

## Sequencing and error correction

Following sequencing of the Chinese hamster, reads were corrected using Illumina reads. To show the correction quality, a sample of 25,000 randomly selected PacBio reads in all three states of correction (raw, after step 1, and final) were mapped to the Illumina contigs. For each read, the indel-ratio (number of indels divided by the number of matches) was computed. The raw reads showed an indel-ratio of 0.18, which could be reduced to 0.06 after the first step and was further reduced to 0.04 in the final step (See Supplementary Figure [S10](#_bookmark2)).

## PacBio SMRT metassembly curation

Assembly using HGAP resulted in a final assembly containing 110,954 contigs with 3.80 Gb total genome sequence. The N50 number was 655 contigs with a N50 length of 995.27 kb. This assembly was about 50% larger than the expected genome size. The weighted histogram of the contig lengths (i.e., the number of contigs, weighted by the total number of bases in the contigs) in Supplementary Figure [S11)](#_bookmark3) shows a clear bimodal characteristic with two peaks at about 20 kb and 3.5 Mb and a local minimum at about 100 kb. The total size of all contigs falling into the second mode (i.e. contigs *≥* 100 kb) is 2.3 Gb (N50 number: 223 and N50 size: 2.9 Mb, see Supplementary Table S1). Realigning the complete assembly back to itself, showed that 80,138 contigs (0.97 Gb) were completely (*≥* 90%) contained in larger contigs (some with rearrangements or larger insertions or deletions). It was expected that these duplicated contigs resulted from poorly corrected reads. Nonetheless, the complete assembly was used for further steps and analyses, since the Metassembler algorithm only merges the best aligning scaffolds (longest alignment, highest percent identity, etc.), while contigs with poorer alignment are discarded. For example, in the first iteration of the merging, about 1.3 Gb were discarded from the PacBio SMRT assembly, while about 20 Mb were discarded from the Illumina assembly.

## Assembly metrics

To identify the best of the eight different assemblies (i.e., four single-platform assemblies and four metassemblies), 80 different metrics were computed, each metric falling in one of six classes. The assemblies were ranked for each metric and the mean rank over all metrics in each class was computed. For the final decision, the mean rank of all class ranks was computed. The assembly with the smallest overall rank was selected as the best overall assembly.

**Class: contig/scaffold numbers**. All common assembly statistics, such as N50, L50, longest contig/scaffold, number of contigs/scaffolds and percentage of gaps in the scaffolds are used as metrics.

**Class: sequence content**. For each pair of two assemblies, one assembly is mapped to the other assembly using NUCmer [Kurtz et al., 2004]. The total number of bases in the first assembly that are not covered by a least one contig of the second assembly is used as a metric of the second assembly. This metric counts the sequence that is missing in the assembly compared to another assembly.

**Class: feature content**. One method to estimate the completeness of an assembly is to count the number of expected features on the genome. Two tools are available that identify the positions of protein-coding genes from a predefined database. The first tool, CEGMA [Parra et al., 2007], searches 248 core eukaryotic genes on the genome and reports the number of complete and fragmented genes. These numbers are used as a metric. A second tool, BUSCO [Simão et al., 2015], uses a similar method. Instead of 248 core genes that could be used for all eukaryotes, BUSCO has a list of genes for different taxonomic levels. The complete and fragmented numbers of the eukaryotic, metazoan and vertebrata were used as metrics. Additionally to these tools, a list of 22,387 mouse coding sequences (CDS) were mapped to the different assemblies using gmap [Wu and Watanabe, 2005] in chimeric mode. A CDS was classified as “complete” if the coverage reported by gmap was greater than 95% and the identity was greater than 75%. If the coverage was below 25% or the identity was below 75% the CDS was classified as “missing”. If gmap reported more than one location for a CDS, it was classified as “chimeric”. The rest was classified as “fragmented”.

**Class: chromosome sorted read coverage**. The chromosome-sorted libraries are mapped to the assemblies using smalt [Genome Research Ltd.,]. The mean coverage of all chromosome libraries is computed for 1 kb regions. The mean coverage of each library is computed by finding the peak in the density plot of all 1 kb regions. This coverage is used to normalize the chromosome coverages for each 1 kb

region. Each region is then classified into five classes bases on the normalized coverages. The first class “low coverage” was applied if the normalized coverage of all chromosomes was below 0.5. The second class “high coverage” was applied, if at least one coverage was above 2. If exactly one normalized coverage was between 0.5 and 2 the 1 kb region was classified as “normal coverage”, and if two or more coverages were between 0.5 and 2, the class “ambiguous” was applied. For each scaffold, the “normal coverage” 1 kb regions were counted for each chromosome. The chromosome with the most 1 kb regions was used as the correct chromosome for this scaffold, while all other “normal coverage” 1 kb regions were reclassified as “false chromosome”.

**Class: remap statistics**. The mappings from the previous class (chromosome sorted read coverage) were also used to compute the percentage of reads that could be mapped back to the scaffolds. All five whole genome paired-end libraries with an insert size of 180 were also included. The libraries were mapped with the same tool and parameter as the chromosome separated libraries. This metric can be used to estimate the completeness of an assembly. The numbers were computed for each library separately.

**Class: CE-statistics**. To identify potential erroneous regions based on CE-statistics and coverage, the tool Reapr [Hunt et al., 2013] was run with pooled mate-pair libraries that showed a similar insert size distribution with a mean of about 5.5 kb. Reapr identifies four kinds of regions, false coverage distribution (FCD) error, FCD error over a gap, low coverage and low coverage over a gap. The false coverage distribution (FCD) error is the difference between the observed coverage of correctly paired mate-pairs to the expected coverage. If this number is below a threshold, the region is reported. If the overall coverage is too low, the region is reported as “low coverage”. The number and total bases of the reported regions were used as metrics.

# Comparison to the mouse and rat genomes

Repeat-masked and unmasked *Mus musculus* and *Rattus norvegicus* genomes were downloaded from Ensembl (Release 86). The repeat-masked PICR scaffolds larger than 1 Mb were mapped to the chromosome sequences of mouse and rat using NUCmer [Kurtz et al., 2004] with –maxmatch option. The delta-filter -1 tool was applied to the mapping results to get the best one-to-one matching of the scaffolds. The results were converted to the DAGchainer input format, and DAGchainer [Haas et al., 2004] was applied to compute chains of consecutive mapping. The unmasked genomes were split at each ‘N’-stretch larger or equal to 100 bases to construct contig sequences for the contig length comparison.

## Contig sizes

Although the *Mus musculus* and *Rattus norvegicus* genomes are available at the chromosome level, they differ at contig level. The mouse assembly consists of 445 contigs (contiguous sequences with N-stretches less than 100 bases), whereas the rat chromosomes are split into 32,025 contigs. The PICR metassembly shows a contiguity between the mouse and the rat assembly, with 4,517 contigs (following splitting contigs at gaps > 100 based to enable consistent comparison between assemblies). The same pattern can be seen with other metrics as shown in Table S9 and in the weighted histogram of contigs length in Figure 1.b.

## Hamster to mouse and rat alignment

The repeat-masked PICR scaffolds larger than 1 Mb were mapped to the repeat-masked mouse and rat chromosomes with NUCmer and the best one-to-one alignment was computed with delta-filter. The alignments then were chained using DAGchainer. The alignment to the mouse chromosomes contained 295,367 single alignments with a mean length of 592.90 bp and a mean identity of 85.39% covering 175.1 Mb (6.65%) of the mouse genome. The chaining created 465 chains with a mean length of 5.0 Mb covering 2.33 Gb (88.74%). The alignment to the rat chromosomes contained 277,685 single alignments with a mean length of 584.40 bp and a mean identity of 85.41% covering 162.2 Mb (5.84%) of the rat genome. The chaining created 1,196 chains with a mean length of 2.0 Mbp covering 2.41 Gb (86.91%). Detailed visualizations are shown in Figures [S12,](#_bookmark4) S13and S14 [for](#_bookmark6) mouse and Figures S15, S16 and [S17](#_bookmark8) for rat alignments.

# Repeat masking, gene prediction, and annotation

## NCBI gene prediction and annotation

The PICR, IPCR, and RefSeq (C_griseus_v1.0, GCF_000419375.1) assemblies were sent to NCBI to undergo a light version of the NCBI annotation pipeline and to gain further insight into the quality of PICR and IPCR regarding gene content. Evidence used in this pipeline included approximately 500 million RNA sequencing reads from CHO cells (SRP066355 [BioProject PRJNA302601] and SRP073484 [BioProject PRJNA318886]). The transcripts and proteins from GenBank and RefSeq for the Chinese hamster, as well as known RefSeq proteins from human and mouse, were used as evidence.

After the evidence was aligned to the assembly sequences, Gnomon [Souvorov et al., 2010] merged overlapping alignments into precursor models. Next, a Hidden Markov Model (HMM) was used to develop *ab initio* gene models and to extend models that were missing a stop or start codon. Gnomon was then run a second time to incorporate alignments of the precursor models to a set of the NCBI nr database. Last of all, some of the resulting gene models were corrected if the evidence from the pipeline strongly suggested that the assembly is wrong.

In addition, external to the pipeline, 30,782 mouse RefSeq transcripts were aligned to PICR, IPCR, and RefSeq. The best sequence alignments were used to determine the number of transcripts that aligned (>= 75% identity) to one scaffold, the number split over two or more scaffolds, the number with no alignments above 75% identity, and the number with no alignments that cover >= 95% of the coding sequences (CDS).

Gene and transcript metrics from the "light" version of the NCBI pipeline are shown in Table S10. The PICR and IPCR assemblies have fewer partial genes, corrected coding region, and genes with premature stops, suggesting that these two assemblies are of higher quality than the RefSeq assembly. Results of the alignment of mouse transcripts from RefSeq are shown in Table S11. PICR has more aligned mouse transcripts than IPCR, which suggests a more complete assembly regarding gene content. While PICR and RefSeq have a similar number of aligned transcripts, PICR has approximately 3 times lower number of split alignments.

# UTR and non-coding assembly analysis

## Blasting of mouse sequences

The mitochondria mediates a variety of metabolic processes that impact maintenance of cellular physiology and homeostasis. Around 1,538 nuclear genes (at 1,654 unique locations) associated with energy metabolism were extracted from *Mus musculus genome* (MM) (assembly GRCm38.p5) and checked for homology within the *Cricetulus griseus* genome (CG) reference assemblies: CSA Cgr1.0 [Brinkrolf et al., 2013], RefSeq C_griseus_v1.0 (criGri1) [Lewis et al., 2013] and the PICR, using NCBI-BLAST-2.6.0+ [Altschul et al., 1990]. These genes comprise of nuclear genes linked to mitochondrial Gene Ontology, components of OXPHOS pathway and the genes in the MitoCarta [Calvo et al., 2015] and QIAGEN (Mouse Mitochondrial Energy Metabolism PCR Array – Version 4.0) lists for mitochondrial energy metabolism.

The analysis was done in three steps to estimate the existence of essential genomic sequences in the three CG assemblies. Primarily keeping the mouse genome as reference, complete genic sequences from TSS-TES were analyzed followed by considering sequences without mouse specific UTR’s (start of the first CDS to the end of the last CDS). The genes reporting homologous regions with the presence of first and last position of the sequence on the same scaffold in respective assemblies were counted using in-house scripts by parsing tabular output of BLASTn reports. For each gene, the hit with first position was flagged as “Start”, last position as “Stop” (split genes) and if both start and end are on the same hit (complete intact gene), it was flagged as “Both”. Keeping the scaffold information along with the flags, for all the genes each of the hit with “Start” flag was checked for the scaffold information in “Stop” flagged hits. If a match was found or if the gene had a hit flagged “Both”, the gene along with its coordinates was noted. All such identified genic locations were then compared for different assemblies and Venn diagrams were plotted.

As maximum genic locations were found complete (split or in single stretch but on the same scaffold) in PICR, it was chosen as reference for the further analysis. These 1,011 genic locations correspond to 948 unique genes in mouse annotation. 858 genes out of these could be found in the PICR annotation at 900 genic locations. Nucleotide sequences were extracted for these 900 locations extending the gene body to include the regulatory regions (5 kb upstream TSS - 1.5 kb downstream TES). These sequences were then analyzed for sequence homology again with the other two CG assemblies to find complete genes along with correctly assembled regulatory regions. For details, see Supplementary Table S6.

The six histone modification marks generated earlier, for PF-MCB CHO-K1 cells sampled twice a day until 9 days, were aligned to the PICR assembly. Based on those alignments, an 11-state model was trained as described before [Feichtinger et al., 2016] and chromatin states were annotated by comparing the emission profiles, displaying enrichment of each histone mark in a state, to the 18-state model deduced from human epigenomes [Kundaje et al., 2015]. Genes annotated on the scaffold margins, with plausibly truncated regulatory information, were identified from the CSA annotation [Brinkrolf et al., 2013], [Feichtinger et al., 2016] and corresponding pattern of chromatin states was observed in both CSA and PICR genomes. Screenshots of examples for genes with the expected promoter or enhancer states found missing in CSA, but clearly observed in PICR, were reported from their respective genome browsers.

# GO term analysis of genes with filled gaps

Gap sequences were identified by aligning flanking sequence from the RefSeq genome to the PICR assembly (Supplementary Figure S9[).](#_bookmark2) To identify which biological processes were overrepresented among the genes with filled gaps, we performed functional GO term analysis of the 2,252 genes using DAVID [Huang et al., 2009b], [Huang et al., 2009a]. The top GO terms are enriched in protein binding, RNA binding, and transcription molecular functions (Supplementary Figure [S6](#_bookmark0)). Gene functional classification results show that these genes are enriched in zinc finger and ribosomal genes. These classes of genes were likely to have filled gaps because they often have highly homologous sequence across the gene families, thus leading to difficulties in resolving their sequence in assemblies based on short reads. In addition, some of the genes locate to repetitive regions in the genome. We further explored which classes of genes had gaps with mutations in several representative resequenced genomes. The top GO terms for these 225 genes are also enriched in DNA binding and transcription (Supplementary Figure [S7](#_bookmark1)). In summary, the gaps in the previous assembly could potentially confound genomic studies in CHO, especially if variants are associated with genes involved in DNA or RNA binding, including transcription factors.

# PIRC to PICR whole genome alignment

Based on the whole genome alignment of PICR and PIRC using NUCmer [Kurtz et al., 2004] and the analysis with dnadiff, 16,305 SNPs or indels and 4,909 structural differences (see show-diff tool) were found. The regions of the PICR assembly with structural differences were combined with all PICR assembly gaps and overlapping regions were merged resulting in 4,130 regions. These regions with the corresponding regions in the PIRC were further analyzed by identifying gaps and possible errors (based on REAPR and read-coverage analysis) in the regions. With this, we could identify:

1. 17 regions with error in the PICR assembly and correct sequence in the PIRC assembly
2. 59 regions with gaps in the PICR assembly and correct sequence in the PIRC assembly
3. 388 regions with gaps in the PICR assembly and error in the PIRC assembly
4. 2,719 regions with gaps in the both assemblies
5. 122 regions with correct sequence in the PICR assembly
6. 165 regions in the PICR assembly without unique corresponding PIRC regions
7. 665 regions with error in the PICR assembly and errors or gaps in the PIRC assembly

Regions a) and b) are possible candidates for error correction in the PICR assembly, with c) gaps in the PICR could be closed but possible errors could be introduced. The corresponding PIRC regions were identified by aligning the bases 300 nt upstream and downstream of the PICR regions to the PIRC scaffolds. Both upstream and downstream regions needed to match uniquely to the scaffolds to identify the regions. Out of these regions, 72 low quality regions in the PICR assembly were manually selected for correction using the PIRC assembly.

Additionally, four candidates for scaffolding were found (based on manual inspection of the alignment). The possible scaffolding order is shown below. The chromosome of the scaffolds is shown in brackets. The PIRC scaffold joining the PICR scaffolds is shown in parentheses.

picr_1193[unplaced] + picr_241[unplaced] (pirc_235)

picr_121[X] + reverse(picr_25[X]) (pirc_16)

reverse(picr_1260[unplaced]) + picr_153[8] (pirc_154)

reverse(picr_653[unplaced]) + picr_167[9] (pirc_168)

A detailed list of the PICR to PIRC alignment with annotated assembly gaps and errors is included in Supplementary Table S12.

**References**

[Altschul et al., 1990] Altschul, S. F., Gish, W., Miller, W., Myers, E. W., and Lipman, D. J., 1990.

Basic local alignment search tool. *Journal of molecular biology*, **215**(3):403–410.

[Brinkrolf et al., 2013] Brinkrolf, K., Rupp, O., Laux, H., Kollin, F., Ernst, W., Linke, B., Kofler, R., Romand, S., Hesse, F., Budach, W. E., *et al.*, 2013. Chinese hamster genome sequenced from sorted chromosomes. *Nature Biotechnology*, **31**(8):694–695.

[Calvo et al., 2015] Calvo, S. E., Clauser, K. R., and Mootha, V. K., 2015. Mitocarta2.0: an updated inventory of mammalian mitochondrial proteins. *Nucleic acids research*, **44**(D1):D1251–D1257.

[Feichtinger et al., 2016] Feichtinger, J., Hernández, I., Fischer, C., Hanscho, M., Auer, N., Hackl, M., Jadhav, V., Baumann, M., Krempl, P. M., Schmidl, C., *et al.*, 2016. Comprehensive genome and epigenome characterization of cho cells in response to evolutionary pressures and over time. *Biotechnology and Bioengineering*, **113**(10):2241–2253.

[Genome Research Ltd., ] Genome Research Ltd. Smalt - sequence mapping and alignment tool v0.7.6.

[Haas et al., 2004] Haas, B. J., Delcher, A. L., Wortman, J. R., and Salzberg, S. L., 2004. DAGchainer: a tool for mining segmental genome duplications and synteny. *Bioinformatics*, **20**(18):3643–3646.

[Huang et al., 2009a] Huang, D. W., Sherman, B. T., and Lempicki, R. A., 2009a. Bioinformatics enrichment tools: paths toward the comprehensive functional analysis of large gene lists. *Nucleic acids research*, **37**(1):1–13.

[Huang et al., 2009b] Huang, D. W., Sherman, B. T., and Lempicki, R. A., 2009b. Systematic and integrative analysis of large gene lists using david bioinformatics resources. *Nature Protocols*, **4**(1):44– 57.

[Hunt et al., 2013] Hunt, M., Kikuchi, T., Sanders, M., Newbold, C., Berriman, M., and Otto, T. D., 2013. REAPR: a universal tool for genome assembly evaluation. *Genome Biology*, **14**(5):R47.

[Kundaje et al., 2015] Kundaje, A., Meuleman, W., Ernst, J., Bilenky, M., Yen, A., Heravi-Moussavi, A., Kheradpour, P., Zhang, Z., Wang, J., Ziller, M. J., *et al.*, 2015. Integrative analysis of 111 reference human epigenomes. *Nature*, **518**(7539):317–30.

[Kurtz et al., 2004] Kurtz, S., Phillippy, A., Delcher, A. L., Smoot, M., Shumway, M., Antonescu, C., and Salzberg, S. L., 2004. Versatile and open software for comparing large genomes. *Genome Biology*, **5**(2):R12.

[Lewis et al., 2013] Lewis, N. E., Liu, X., Li, Y., Nagarajan, H., Yerganian, G., O’Brien, E., Bordbar, A., Roth, A. M., Rosenbloom, J., Bian, C., *et al.*, 2013. Genomic landscapes of Chinese hamster ovary

cell lines as revealed by the Cricetulus griseus draft genome. *Nature Biotechnology*, (8):759-765.

[Parra et al., 2007] Parra, G., Bradnam, K., and Korf, I., 2007. CEGMA: a pipeline to accurately annotate core genes in eukaryotic genomes. *Bioinformatics*, **23**(9):1061–1067.

[Simão et al., 2015] Simão, F. A., Waterhouse, R. M., Ioannidis, P., Kriventseva, E. V., and Zdobnov,

E. M., 2015. BUSCO: assessing genome assembly and annotation completeness with single-copy orthologs. *Bioinformatics*, **31**(19):3210–3212.

[Souvorov et al., 2010] Souvorov, A., Kapustin, Y., Kiryutin, B., Chetvernin, V., Tatusova, T., and Lipman, D., 2010. Gnomon–NCBI eukaryotic gene prediction tool. *National Center for Biotechnology Information*, 1–24.

[Wu and Watanabe, 2005] Wu, T. D. and Watanabe, C. K., 2005. GMAP: a genomic mapping and alignment program for mRNA and EST sequences. *Bioinformatics*, **21**(9):1859–1875.
